# The effect of a one-year vigorous physical activity intervention on fitness, cognitive performance and mental health in young adolescents: the Fit to Study cluster randomised controlled trial

**DOI:** 10.1186/s12966-021-01113-y

**Published:** 2021-03-31

**Authors:** T. M. Wassenaar, C. M. Wheatley, N. Beale, T. Nichols, P. Salvan, A. Meaney, K. Atherton, K. Diaz-Ordaz, H. Dawes, H. Johansen-Berg

**Affiliations:** 1 Wellcome Centre For Integrative Neuroimaging, FMRIB, Nuffield Department of Clinical Neurosciences, University of Oxford, John Radcliffe Hospital, Headley Way, Oxford, OX3 9DU UK; 2 Department of Sport Health Sciences and Social Work, Centre for Movement Occupational and Rehabilitation Sciences, Oxford Brookes Centre for Nutrition and Health, Oxford Brookes University, Oxford, OX3 0BP UK; 3 Oxford Big Data Institute, Li Ka Shing Centre for Health Information and Discovery, Nuffield Department of Population Health, University of Oxford, Oxford, OX3 7LF UK; 4 Department of Medical Statistics, London School of Hygiene and Tropical Medicine, Keppel Street, London, WC1E 7HT UK

**Keywords:** Physical activity, Adolescence, Cognition, Mental health, Cardiorespiratory fitness, Cluster randomised controlled trial, Intervention

## Abstract

**Background:**

Physical activity (PA) may positively stimulate the brain, cognition and mental health during adolescence, a period of dynamic neurobiological development. High-intensity interval training (HIIT) or vigorous PA interventions are time-efficient, scalable and can be easily implemented in existing school curricula, yet their effects on cognitive, academic and mental health outcomes are unclear. The primary aim of the Fit to Study trial was to investigate whether a pragmatic and scalable HIIT-style VPA intervention delivered during school physical education (PE) could improve attainment in maths. The primary outcome has previously been reported and was null. Here, we report the effect of the intervention on prespecified secondary outcomes, including cardiorespiratory fitness, cognitive performance, and mental health in young adolescents.

**Methods:**

The Fit to Study cluster randomised controlled trial included Year 8 pupils (*n* = 18,261, aged 12–13) from 104 secondary state schools in South/Mid-England. Schools were randomised into an intervention condition (*n* = 52), in which PE teachers delivered an additional 10 min of VPA per PE lesson for one academic year (2017–2018), or into a “PE as usual” control condition. Secondary outcomes included assessments of cardiorespiratory fitness (20-m shuttle run), cognitive performance (executive functions, relational memory and processing speed) and mental health (Strength and Difficulties Questionnaire and self-esteem measures). The primary intention-to-treat (ITT) analysis used linear models and structural equation models with cluster-robust standard errors to test for intervention effects. A complier-average causal effect (CACE) was estimated using a two-stage least squares procedure.

**Results:**

The HIIT-style VPA intervention did not significantly improve cardiorespiratory fitness, cognitive performance (executive functions, relational memory or processed speed), or mental health (all *p* > 0.05). Subgroup analyses showed no significant moderation of intervention effects by sex, socioeconomic status or baseline fitness levels. Changes in cardiorespiratory fitness were not significantly related to changes in cognitive or mental health outcomes. The trial was marked by high drop-out and low intervention compliance. Findings from the CACE analysis were in line with those from the ITT analysis.

**Conclusion:**

The one-academic year HIIT-style VPA intervention delivered during regular school PE did not significantly improve fitness, cognitive performance or mental health, but these findings should be interpreted with caution given low implementation fidelity and high drop-out. Well-controlled, large-scale, school-based trials that examine the effectiveness of HIIT-style interventions to enhance cognitive and mental health outcomes are warranted.

**Trial registration:**

ISRCTN registry, 15,730,512. Trial protocol and analysis plan for primary outcome prospectively registered on 30th March 2017.

ClinicalTrials.gov, NCT03286725. Secondary measures (focus of current manuscript) retrospectively registered on 18 September 2017.

**Supplementary Information:**

The online version contains supplementary material available at 10.1186/s12966-021-01113-y.

## Background

Adolescence is marked by rapid, dynamic social, psychological and neurocognitive development, during which the brain matures and higher-order cognitive processes, such as executive functions, are refined [1–3]. It is also the peak onset time for mental health issues [4, 5], including an increased social and biological vulnerability to mood disorders and low self-esteem. Notably, adolescence has been described as a (neurobiological) critical or sensitive period of development [2, 6], characterised by enhanced plasticity, during which experience or environmental influences strongly affect the brain and may have long-lasting effects on behaviour. This period in life therefore presents an opportunity for interventions to have a positive impact on short- and long-term health outcomes, and has been of increasing interest to researchers and policymakers [1, 7].

Interventions involving regular physical activity (PA) have received much attention given that PA is easily accessible, modifiable, cost-effective, scalable and has well-established health benefits, including improvements in cardiometabolic health [8]. Converging lines of evidence suggest that regular PA changes the brain by stimulating processes such as neurogenesis and angiogenesis [9], and could improve cognitive and mental health in young people [10]. In particular, intervention studies involving primarily pre-adolescent children have found some evidence that PA improves cognition, particularly in the domains of attention and executive functions (working memory, cognitive flexibility and inhibitory control), as well as attainment in maths, but considerable between-study heterogeneity makes firm conclusions difficult to draw [11–14]. A smaller body of work suggests PA can treat depression, reduce anxiety, and improve self-esteem [10, 15]. There is also emerging evidence that childhood PA is predictive of cardiovascular, mental, cognitive, and brain health later in life [16–19]. However, approximately 80% of adolescents do not meet the recommended 60 min of moderate-to-vigorous PA (MVPA) per day [20, 21] and PA levels decline dramatically during teenage years [22]. Hence, there is scope for interventions to increase PA levels.

Schools are an ideal setting to promote PA among adolescents, including those from lower socioeconomic backgrounds, because pupils spend a large proportion of their waking hours at school and schools have access to the required facilities [23]. Findings from school-based PA interventions have been inconsistent, with recent reviews reporting no meaningful change in PA across the (school) day [24, 25], but enhanced (moderate-to-vigorous) PA during the actual intervention [25, 26]. More promising results have been reported for school-based interventions aimed to increase cardiorespiratory fitness (CRF) [27–30], self-esteem [31], and cognitive performance, with meta-analyses showing that interventions delivered during curricular physical education (PE) are particularly effective at improving cognition [13, 14]. The majority of these interventions, however, have involved large doses of PA (e.g. 60 min 4 times per week) that may not be easily implemented in, and scaled up to, all schools, where crowded school curricula require PA interventions to be time-efficient [32, 33].

High-intensity interval training (HIIT) is a time efficient and cost-effective strategy to improve cardiovascular health and CRF in young people [34–37] that can be implemented easily during the school day [38]. HIIT definitions vary [39], but typically involve short or longer bouts (from < 45 s to 2–4 min) of high-intensity exercise interspersed by periods of rest or light activity [40, 41], leading to physical adaptations that are comparable to endurance training results [42, 43]. While interventions involving working at or near the maximum intensity (e.g. > 90% VO_2_peak, or max heart rate) may be limited in their scalability and effectiveness [39], there is some promising evidence from less intense HIIT interventions showing improvements in executive functions [44–49] and mental well-being [45, 49, 50]. However, most of these interventions were brief (6–14 weeks), relatively well-controlled with small samples (1–6 schools), and did not investigate academic outcomes. The “Burn 2 Learn” cluster randomised trial, which delivered HIIT activity breaks twice a week for six months during curriculum time in 10 intervention schools (compared to 10 control), showed a significant improvement in older adolescents’ (mean age = 16) CRF at six, but not 12 months. In contrast to previous studies, however, it observed no significant improvements in cognitive and mental health outcomes [37, 51]. More pragmatic and large-scale trials, including those involving younger adolescents, are needed to assess the effectiveness and scalability of HIIT.

The Fit to Study cluster randomised trial investigated the effect of a one- academic year HIIT-style intervention delivered by PE teachers during regular PE on maths attainment, mental health, cognitive performance and CRF in young adolescents (12–13 years old) from 104 schools (52 intervention) in South/Mid England [52]. An independent evaluator, NatCen Social Research, analysed the primary outcome, attainment in maths, and found no significant benefit of the HIIT-style intervention [53]. Here, we report the prespecified secondary outcomes of the Fit to Study cluster RCT. We hypothesised that the scalable HIIT-style intervention would improve CRF, cognitive performance and mental health outcomes in this cohort. We also explored whether the effect of the intervention on the prespecified outcomes was moderated by sex, socioeconomic status, or baseline CRF level. We additionally examined whether changes in CRF were related to changes in cognitive and mental health outcomes.

## Methods

The reporting of this trial followed the Consolidated Standards of Reporting Trial guidelines (CONSORT; checklist is provided in Additional File 1). The template for intervention description and replication (TIDieR) checklist is provided in Additional File 2.

### Design

The Fit to Study project was a parallel group, superiority cluster-randomised efficacy trial of a one academic year HIIT-style VPA intervention versus control involving ﻿Year 8 pupils (aged 12–13 years) from 104 secondary state schools (52 intervention). The intervention was incorporated into regular PE lessons to minimally disrupt the curriculum and ensure scalability. Assessments took place at baseline (end of Year 7, pupils aged 11–12 years) and after 12 months (end of Year 8, pupils aged 12–13 years).

The trial was approved by the Central University Research Ethics Committee of Oxford University (Registration No. R48879). The trial protocol, including analysis plans for the primary outcome measure (attainment in maths), was prospectively registered on the ISRCTN registry (15730512). The protocol of the secondary measures was retrospectively registered at ClinicalTrials.gov (NCT03286725, 18 September 2017).

Full details of the study design and secondary measures are available elsewhere [52]. The primary outcome was analysed by an independent evaluator, NatCen Social Research, and published by the Education Endowment Foundation [53].

### Sample

The National Foundation for Educational Research (NFER) led recruitment, supported by Oxford Brookes University. Eligible schools could be either mixed or single sex secondary state or academy schools in South/Mid-England that delivered PE as part of their curriculum, with a proportion of pupils eligible for free school meals (eFSM), preferably more than 15%, which was the average for England at the time of recruitment. Their Year 7 pupils had to move on to Year 8 at the start of the intervention, and they had to be willing to sign an agreement to send opt-out consent forms to parents/carers of Year 7 pupils. A total of 106 eligible schools responded to an invitation to participate in the study. Following the eligibility assessment, two schools declined to participate, and, from those remaining, 81 Year 7 pupils opted out of data storage and were not required to complete the trial’s assessments. Schools that met inclusion criteria and wished to participate provided the sex, age and eFSM status of all Year-7 pupils; they were considered formally recruited upon transfer of the data and memorandum of understanding to NFER. A total of 104 schools (*n* = 18,261 pupils) were randomised into an intervention and control group.

Measures of self-reported physical activity in the past week (0–7 days) [54] and habitual physical activity over the past week (range 1–7, with 7 the most active) [55] were used to characterise the sample’s baseline PA levels.

### Randomisation and blinding

Schools (*n* = 104) were allocated to an intervention group or “PE as usual” control group (1:1 ratio) using stratified block randomisation. Stratification was arranged according to whether schools were single-sex or co-educational. NatCen Social Research performed the randomisation using a random number generator in Stata (version 12) [56].

Schools were informed of their assigned group following baseline assessments to minimise bias and so that intervention PE teachers could receive training and deliver the intervention. Given the nature of the intervention, it was not feasible for PE teachers or pupils to be blind to group allocation, but neither pupils nor parents were specifically told their assigned group by the research team. Researchers visiting schools for top-up training and collection of fidelity measures were not blinded.

### Intervention

The intervention, co-developed with PE experts and teachers, consisted of a one academic year (10 months: September 2017–June 2018) HIIT-style VPA programme delivered by PE teachers during regular Year 8 PE lessons. Government guidance suggests a minimum of 2 h per week of curricular PE, which is typically scheduled in two, one-hour lessons. Teachers were instructed to incorporate two elements of additional VPA bursts into lessons, consisting of: (1) 4 min of VPA as part of an active 10-min warm-up, and (2) three 2-min (VPA) infusions per hour of PE, where VPA was defined as activity that raises the heart rate to 71–85% of the maximum heart rate [57]. We defined the intervention as HIIT-*style* because it meets several HIIT criteria [40, 41], yet other HIIT definitions exist that emphasise a higher intensity [58]. To minimise the risk of injuries, the teacher training stressed that pupils should be working vigorously rather than maximally.

The intervention was co-developed with PE experts and teachers. A pilot phase in seven schools explored the feasibility of a multi-component approach to maximising activity during PE, which included a mix of practical lesson organisation strategies and theory-led teaching principles [59]. But feedback from schools indicated a simpler, more structured intervention would be more feasible for teachers to administer while also following the PE curriculum. The efficacy of brief fitness “infusions” to raise heart rate was confirmed in pilot schools. While acknowledging the usefulness of implementation frameworks for optimising study design for disparate settings, we concluded that a simple intervention was best suited to our range of geographical, social and cultural environments.

The warm-up was delivered at the start of each lesson and began with light-intensity movements (e.g. wrist rotations), followed by moderate-intensity activities (e.g. arm rotations) to incorporate two 2-min bursts of VPA (e.g. vigorous arm sprints; running on the spot). The three 2-min infusions (per hour of PE) were incorporated in the main PE lesson. The infusions included, for instance, fast arm rotations, squats and lunges, and sprinting on the spot, interspersed with brief (active) rest periods if needed during each infusion. These were intended to improve CRF, with some incidental benefits for muscular fitness. Teachers were invited to create their own warm-up and/or infusions that met the required intensity and duration of PA.

Schools in the control arm were asked to deliver PE as usual. To minimise drop-out and ensure compliance, school participation was incentivised with each school receiving £500 upon completion of the primary outcome assessment post-intervention.

### Teacher training

PE teachers from intervention schools were trained prior to the start of the intervention by attending an online or face-to-face 2-h training session delivered by a team from Oxford Brookes University. Sessions provided an overview of VPA and guideline daily activity levels, how increasing PA is thought to benefit learning and thinking skills, an overview of intervention components and a timetable of assessments in school. Sessions also included a practical exercise on lesson planning and videos of an experienced PE teacher delivering infusions. Following the training session, teachers from intervention schools were given access to the trial’s website containing videos demonstrating the intervention elements. Researchers visited a sample of 30 intervention schools between December 2017 and February 2018 to offer support and answer questions. They offered additional support and top-up training to PE teachers throughout the intervention period.

### Intervention fidelity

Intervention fidelity was assessed using a prespecified set of measures, including (1) teacher log books collected throughout the year in intervention schools, (2) an objective measure of class-average minutes of VPA per hour of PE using wrist-worn Axivity AX3 tri-axial accelerometers (Open Lab, Newcastle University, UK) in a convenience sample of participants per school (at least 50% of the Year group, collected once during the intervention period; data processing details are provided in Additional file 3), and (3) pupil-reported compliance with the intervention using a brief three-question survey administered post-intervention in all schools asking whether (i) PE lessons started with a warm-up, (ii) PE lessons included bursts of VPA that raised their heart rate and made them feel out of breath, and (iii) they took part in warm-ups or VPA bursts if asked by PE teachers. Due to low compliance with the log books, NatCen Social Research additionally collected the teacher-reported percentage of PE lessons in which the intervention was delivered as intended (post-intervention, in intervention schools only), as part of the trial evaluation.

### Outcome measures

Pupils were assessed before the intervention started (pretest, t_0_, end of Year 7 prior to the summer break) and immediately following the intervention (posttest, t_1_). Secondary measures included assessments of CRF, cognitive performance, and mental health.

#### Cardiorespiratory fitness

CRF was assessed using the 20-m shuttle run (20MSR) test [60]. The outcome measure was the total number of laps completed. Schools with a policy of not using the 20MSR completed the 12 min Cooper Run test instead (*n* = 4, [61]).

#### Cognitive performance

Cognitive functioning was assessed with online, computer-based tests of processing speed, visual relational memory and core executive functions (working memory, inhibition, and cognitive flexibility) [62]. The cognitive assessments were programmed in JavaScript using jsPsych [63] and took approximately 50 min to complete. The task order was pseudorandomised across participants within schools. Each participant started with the reaction time task, followed by the remaining tasks in random order. Teachers and pupils were instructed to complete the assessments at home, but a proportion of schools decided to complete the tasks during school time.

Details of the cognitive battery have been provided in the protocol [52]. In brief, the battery consisted of the following tasks:

- A simple reaction time task was used to assess processing speed. The average reaction time across valid trials was the primary outcome measure.
- A modified version of a previously described relational memory task [64] was used to assess visual relational memory performance. The proportion of correct responses on valid trials (i.e. accuracy) was the primary outcome measure.
- A modified Flanker task [65] was used to assess inhibitory control. The congruent and incongruent response accuracies were the primary outcome measures.
- A visual two-back task [66, 67] was used to assess working memory performance. Response accuracy was the primary outcome measure.
- A modified colour-shape switching task [65] was used to assess cognitive flexibility. The switch and non-switch response accuracies were the primary outcome measures.

#### Mental health

Psychosocial problems were measured with the Strength and Difficulties Questionnaire (SDQ) [68], which consists of 25 items measuring five sub-scales: (1) emotional symptoms, (2) conduct problems, (3) hyperactivity / inattention, (4) peer-relationship problems, (5) pro-social behavior. Items are scored from 0 (“not true”) to 2 (“certainly true”). We used combined conduct and hyperactivity scores (externalising, range 0–20) and peer and emotional scores (internalising, range 0–20) as primary outcome measures because there is evidence that in low-risk samples, the more focused subscales might not tap into distinct aspects of mental health [69].

Self-esteem was assessed with the global and physical self-esteem subscales of the short version of the Physical Self-Description Questionnaire [70] (range: 1–7; higher scores indicate better outcomes).

### Data analysis

#### Sample size

Sample size calculations were performed by NatCen for the primary intention-to-treat (ITT) analysis, which compared maths test performance between intervention and control schools [56]. Details on the sample size calculation, including an *a-posteriori* computed minimum detectable effect size for CRF, cognitive- and mental health outcomes, are provided in Additional file 3.

#### Data cleaning

The computer-based questionnaire and cognitive assessments were completed in school or at home, without supervision from researchers or teachers. A conservative approach to data cleaning was used, that focussed on removing careless responders or data collection errors from the dataset. We additionally removed schools (*n* = 4, 561 pupils) that completed the Cooper Run Test. Details of the cleaning procedures are provided in Additional file 3.

#### Statistical analysis

The primary analyses were performed on an intention-to-treat basis on multiply imputed data under the assumption data was missing at random (MAR). To assess the effect of the intervention on CRF, cognitive outcomes, and mental health, we followed recommendations for (cluster) randomised trials: models included baseline values of the outcome measure (ANCOVA) and included the stratification variable used at randomization (school gender-type) [71]. Standard errors were corrected for the clustering of pupils within school using robust (sandwich) estimators (type CR2), that have shown good performance even with a small number of clusters [72].

The primary analyses assessed the effect of the intervention on CRF, mental health (self-esteem and internalising-, and externalising symptoms) and cognitive performance. We used confirmatory factor analysis to create an executive function (EF) latent defined by accuracy measures of the colour-shape switching task, Flanker task and two-back task. We used a latent variable model to capture the common variance shared by these different measures. Structural equation models (SEM) were constructed with Full Information Maximum Likelihood estimators to allow for missing data. Standard errors were adjusted for clustering (Huber-White). We subsequently additionally adjusted the primary analysis models for the period *(*i.e. summer term, holidays or autumn term) and location (i.e. school or home) the tasks were completed.

In line with the analysis of the primary outcome [53], exploratory subgroup analyses were conducted to assess whether any effect of the intervention on secondary outcomes (EF latent variable, relational memory, processing speed, in−/externalising symptoms and global/physical self-esteem) was moderated by sex, socioeconomic status or high or low baseline CRF. Baseline CRF levels were dichotomised into high- and low-fit category using sex-specific normative values (high fit males > 39 laps, high fit females > 28 laps) [73].

We also explored whether a change in CRF was related to a change in cognitive measures or mental health, and whether this relationship was moderated by treatment status, controlling for age, sex, socioeconomic status (eFSM), school gender-type (single sex or co-educational), and location (home or school) and period of completion (summer term, holidays or autumn term) of assessments, using linear models with cluster-robust standard errors.

The primary ITT analysis will provide a conservative (or underestimated) treatment effect when schools do not adhere to the intervention [74]. We therefore estimated a complier average causal effect (CACE) by fitting an instrumental variable model using the two-stage least squares method, to estimate the average treatment effect in the population of compliers (details are provided in Additional File 3). Intervention schools in which the intervention was delivered in > 50% of PE lessons were classified as compliant.

We used multilevel multiple imputation by chained equations (MICE) to impute missing data under the assumption that data was MAR (details in Additional files 3 and 4). We separately imputed the data for the CACE analyses, given the large amount of missingness in the fidelity measure. As a sensitivity analysis, we repeated all the analyses with complete-case data (i.e. available cases per analysis model).

Two-sided inferences with *p* < 0.05 were considered statistically significant. The adjusted standardized and unstandardized mean differences are provided. The standardized mean difference score was computed using the pooled unconditional standard deviation of the outcome [53, 75]. Bonferroni-correction was applied to correct for multiple comparisons where appropriate. An overview of analysis software is presented in Additional file 3.

## Results

**Fig. 1:**
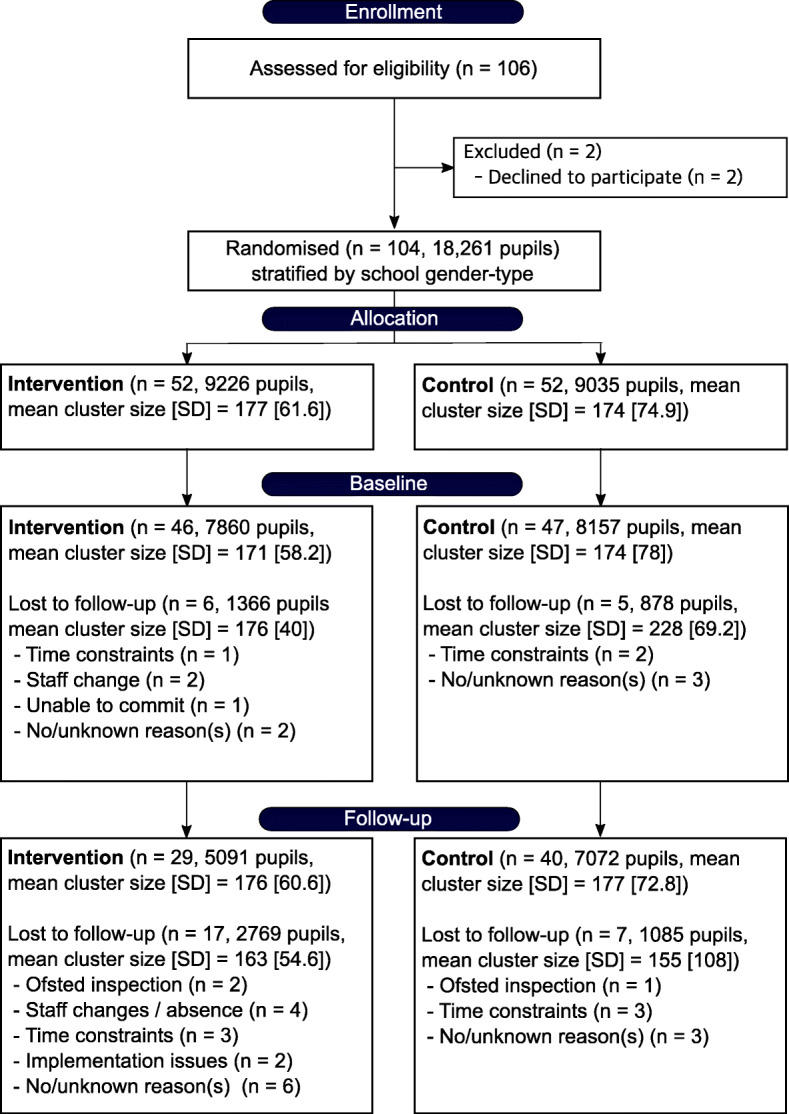
CONSORT flow diagram of schools and participants

One hundred-four schools, totalling 18,261 pupils participated in the study (Fig. 1), and were randomised into an intervention and control group. Prior to baseline assessments but post-randomisation, 11 schools withdrew from the trial. The schools were unaware of their allocated group at the time of drop-out and school or pupil level demographics did not significantly predict drop-out (Additional file 5). Analyses were therefore limited to the schools that were part of the trial at baseline assessments (*n* = 93, 16,017 pupils). A total of 24 schools (17 intervention) were further lost to follow-up.

The trial intended to collect all secondary outcomes in all participants, but for various reasons, including both school drop-out and non-completion at the level of individual pupils, some degree of missing data was present for all secondary measures. Out of 16,017 pupils at baseline, 2182 (~ 14%) completed all secondary outcomes, 8114 (~ 51%) completed the CRF assessment, 6414 (~ 40%) completed the questionnaire and 6174 (~ 39%) completed one or more cognitive assessments, following data cleaning, at post-test. Excluded participants during data cleaning were more likely to be male and from a lower socioeconomic background (see Additional file 6 for an overview). The primary analyses were performed on an intention-to-treat basis on multiply imputed data (*n* = 16,017). Sensitivity analyses using complete-cases only are reported at the end of the results section.

### Sample characteristics

Descriptive statistics for school- and pupil level characteristics at baseline (*n* = 93) are presented in Table 1 (additional school characteristics are presented in additional file 7). The groups are approximately similar on all measures.

**Table 1:** School- and pupil level baseline characteristics

	Intervention	Control
**School level**		
No. schools	46	47
Gender status, no. (%)		
Co-ed	37 (80.4%)	38 (80.9%)
Female	9 (19.6%)	8 (17.0%)
Male	0 (0%)	1 (2.1%)
Socioeconomic status		
Percent eFSM pupils, mean (SD)	17.0 (9.00)	17.7 (12.9)
IMD, median (range), decile	5 (1–10)	6 (1–10)
**Pupil level**		
No. pupils	7860	8157
Age, mean (SD), y	12.5 (0.296)	12.5 (0.293)
Females, no (%)	4466 (56.8%)	4495 (55.1%)
eFSM, no. (%), yes	1243 (15.8%)	1422 (17.4%)

*Abbreviations*: *Co-ed* co-education, *eFSM* eligible for free school meals, *IMD* index of multiple deprivation, *SD* standard deviation, *y* year

Approximately 20% of the sample reported that they were physically active for at least 60 min per day for the entire week (i.e. meeting the PA guidelines), which is comparable between the groups (Additional file 7). Moreover, across schools, on average, 14 min per hour of PE was spent in MVPA, and approximately 4 min in VPA.

School curricula in intervention and control schools included, on average, a similar number of PE lessons (1.61 vs 1.59, range: 1–2), a comparable duration of PE lessons (72.3 vs 72.2 min), and total duration of PE per week (110 vs 107 min; details in Additional file 7).

### Effects on cardiorespiratory fitness

CRF levels increased from baseline to post-test across groups (Table 2, and distribution plots of complete-cases in Additional file 8). However, no significant difference in CRF levels were observed between the intervention and control group at posttest (standardized mean difference [SMD] = 0.02, 95% CI: -0.11, 0.16).

**Table 2:** Mean scores for outcome measures by group, with (un)standardized mean differences

	Intervention group (46 schools, ***n*** = 7860)		Control group (47 schools, ***n*** = 8157)		Adjusted mean difference^**1**^ (95% CI)	
	Baseline	Post	Baseline	Post	Unstandardized	Standardized^2^
	M (SD)	M (SD)	M (SD)	M (SD)		
20MSR						
Fitness, laps	36.11 (20.75)	41.36 (21.95)	37.85 (20.65)	42.37 (22.28)	0.48 (−2.52, 3.48)	0.02 (−0.11, 0.16)
Reaction time task						
RT^3^, ms	378.7 (86.65)	378.58 (99.46)	380.21 (89.7)	376.07 (95.94)	3.63 (−7.04, 14.3)	0.04 (−0.07, 0.15)
Relational memory task						
Accuracy, %	59.75 (11.93)	59.53 (13.05)	59.76 (12.24)	60.78 (13.08)	−1.37 (−3, 0.26)	−0.1 (−0.23, 0.02)
Two-back task						
Accuracy, %	54.43 (18.46)	55.24 (20.9)	53.4 (18.74)	55.48 (20.99)	−0.96 (−3.38, 1.47)	−0.05 (−0.16, 0.07)
RT^3^, ms	857.67 (164.84)	802.26 (175.03)	844.17 (171.04)	799.45 (174.9)	−4.25 (−23.09, 14.58)	−0.02 (−0.13, 0.08)
Flanker task						
Accuracy congruent, %	82.83 (17.14)	84.21 (16.57)	81.91 (16.81)	84.55 (16.44)	−0.92 (−2.38, 0.53)	−0.06 (−0.14, 0.03)
Accuracy incongruent, %	60.22 (20.67)	62.2 (20.33)	60.02 (19.55)	63.06 (19.65)	−1.05 (−2.86, 0.76)	−0.05 (−0.14, 0.04)
RT^3^ congruent, ms	490.26 (92.13)	476.82 (84.22)	490.35 (94.51)	479.19 (81.26)	−2.49 (−10.37, 5.39)	−0.03 (−0.13, 0.07)
RT^3^ incongruent, ms	552.9 (127.12)	537.89 (108.67)	548.15 (126.16)	541.25 (109.06)	−5.74 (−15.41, 3.93)	−0.05 (−0.14, 0.04)
Colour-shape switching task						
Accuracy non-switch, %	73.27 (17.21)	75.18 (17.61)	72.88 (17.25)	75.61 (17.48)	−0.76 (−2.64, 1.12)	−0.04 (−0.15, 0.06)
Accuracy switch, %	69.51 (16.89)	71.47 (17.65)	69.36 (17.15)	71.56 (17.59)	−0.32 (−2.16, 1.52)	−0.02 (−0.12, 0.09)
RT^3^ non-switch, ms	1075.67 (352.18)	974.95 (320.46)	1051.07 (337.52)	970.87 (318.08)	−5.86 (−34.33, 22.61)	−0.02 (−0.11, 0.07)
RT^3^ switch, ms	1452.99 (602.23)	1331.63 (562.05)	1404.21 (569.5)	1321.97 (544.16)	−13.18 (−66.27, 39.91)	−0.02 (−0.12, 0.07)
Psychosocial problems						
Internalising score^3^	5.1 (3.56)	5.47 (3.72)	5.07 (3.49)	5.25 (3.6)	0.19 (−0.18, 0.57)	0.05 (−0.05, 0.16)
Externalising score^3^	6.34 (3.85)	6.73 (3.78)	6.29 (3.74)	6.45 (3.8)	0.24 (−0.13, 0.61)	0.06 (−0.03, 0.16)
Self-esteem						
Global	4.39 (0.94)	4.23 (1.03)	4.43 (0.9)	4.3 (0.97)	−0.05 (−0.22, 0.12)	−0.05 (−0.22, 0.12)
Physical	4.36 (1.31)	4.07 (1.41)	4.44 (1.27)	4.14 (1.36)	−0.02 (−0.22, 0.19)	−0.01 (−0.16, 0.14)

*Abbreviations*: *CRF* cardiorespiratory fitness, *ms* millisecond, *RT* reaction time

^1^ Adjusted mean difference, adjusted for clustering, baseline values of the outcome variable and school gender-type

^2^ The outcome was standardized (mean = 0, SD = 1), prior to fitting the baseline and stratification-variable adjusted model

^3 ^Lower scores represent better performance

### Effects on cognitive function

**Fig. 2:**
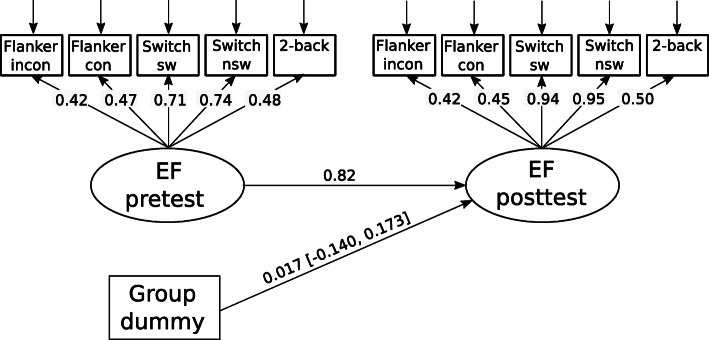
Path diagram showing the effects of the VPA intervention on an Executive Function (EF) latent variable. All coefficients are standardized except for the path coefficient for Group which is y-standardized (equivalent to Cohen’s *d*). The 95% robust (Huber-White) confidence interval (CI) is shown for the Group coefficient. The stratification variable (school gender-type, dummy coded) was included as a set of additional covariates but they are not shown in the model. The following correlations between residuals were included in the model (t_0_ = pretest, t_1_ = posttest): Flanker_incon, t0_ with Flanker_incon,t1_ (0.34), Flanker_con,t0_ with Flanker_con, t1_ (0.29), Switch_nsw, t0_ with Switch_nsw, t1_ (0.09), Flanker_con, t0_ with Flanker_incon, t0_ (0.39) Switch_nsw, t0_ with Switch_sw, t0_ (0.074), Flanker_incon, t1_ with Flanker_con, t1_ (0.41), 2-back_t1_ with 2-back_t0_ (0.38), but are not shown in the diagram. Abbreviations: EF = executive function, incon = incongruent, con = congruent, nsw = non-switch, sw = switch

The primary cognitive outcome measure was an EF latent variable defined by accuracy measures of the colour-shape switching task, Flanker task and two-back task. We used a latent variable model to capture the common variance shared by these different measures. The model used is shown in Fig. 2 and provides an adequate fit to the data (χ^2^ (54) = 739.431; *p* < 0.001; RMSEA [Root Mean Square Error of Approximation] = 0.037 [90% CI: 0.035, 0.039]; CFI [Comparative Fit Index] = 0.97; TLI [Tucker-Lewis Index] = 0.96). The EF latent variable shows considerable longitudinal stability (standardized coefficient = 0.82) demonstrating that this construct has good reliability, despite a lack of factorial invariance (i.e. the factor loadings on the EF factor vary across time), possibly because of a differential change in individual assessments due to the intervention. The intervention group showed no significant difference in EF skills compared to the control group at posttest (SMD = 0.017, 95% CI:  -0.14, 0.17).

An improvement in relational memory performance and processing speed (reaction time task) was observed across groups from pre to post-intervention. However, no significant difference was observed in relational memory performance (SMD =  -0.1, 95% CI:  -0.25, 0.02) or processing speed (SMD = 0.04, 95% CI:  -0.07, 0.15) between the intervention and control group at posttest.

### Effects on mental health

Participants reported an increase in psychosocial problems from pre to post-intervention. The primary ITT analysis, however, demonstrated no significant difference in internalising (SMD = 0.05, 95%CI:  -0.05, 0.16) and externalising scores (SMD = 0.06, 95%CI: -0.03, 0.16) between intervention and control groups at posttest. Moreover, across treatment groups, a decrease in global- and physical self-esteem was observed from baseline to posttest. However, pupils in the intervention group did not differ significantly in their global (SMD =  -0.05, 95%CI:  -0.22, 0.12) or physical self-esteem (SMD =  -0.01, 95%CI: − 0.16, 0.14) compared to control at posttest.

### Subgroup analysis

The effect of the intervention on cognitive and mental health outcomes was not significantly moderated by participants’ sex, socioeconomic status or baseline CRF levels, following correction for multiple comparisons (alpha = 0.002, with 25 comparisons; details in Additional File 9). Uncorrected analyses showed that two (of 25) comparisons had a *p*-value just below the conventional significance threshold (at *p* = 0.04, and *p* = 0.03).

### Relationship of change in CRF with changes in cognition and mental health

One-year changes in CRF were not significantly related to changes in cognitive measures or indicators of mental health (internalising-, externalising symptoms, or self-esteem; all corrected-*p* > 0.05, details are provided in Additional file 10), and these relationships were not moderated by treatment group (corrected-*p* > 0.05 for interactions).

### Intervention fidelity and complier average causal effect analysis

School-level and pupil level fidelity measures were collected towards the end of the intervention or post-intervention (details in Additional file 11). Of 46 intervention schools at baseline, 15 (33%) received face-to-face training, 26 (57%) attended an online training programme and 5 (11%) did not receive training (and were lost to follow-up). Objective measures of PA collected with accelerometers during a single PE lesson during the intervention indicated that pupils in intervention schools spent, on average, more lesson time in VPA compared to control (full lesson: 3.65 vs 3.09 min/hour, *p* = 0.23; active lesson: 4.91 vs 4.38 min/hour, *p* = 0.07), but this difference was not statistically significant. Pupil-reported measures of compliance suggested that PE lessons in both intervention and control schools often started with a warm-up and incorporated infusions (median = 4, with 1 = “never” and 5 = “always”). Moreover, pupils in both treatment and control schools indicated that they would take part in a warm-up and infusions if asked by a PE teacher (median: 4.75 vs 5, respectively).

Of 29 intervention schools that were part of the trial at follow-up, 22 provided information on the percentage of PE lessons in which the intervention was delivered as intended. Of these schools, 17 (77%) reported having delivered the intervention as intended in at least 50% of PE lessons across the year and only two schools (9%) delivered the intervention in over 90% of PE lessons. CACE estimates were similar in directionality to and in line with those obtained from the ITT analysis, and are provided in Additional file 11.

### Additional analyses

We inspected whether there were baseline differences in self-reported PE enjoyment and attitudes towards PA between the groups, which could make the groups more or less susceptible to intervention effects. There was no evidence of a difference in PE enjoyment (*p* = 0.91) or PA attitudes at baseline (*p* = 0.57, details in Additional file 12). Moreover, no significant differences were observed in PE enjoyment (*p* = 0.58) or attitudes towards PA (*p* = 0.1) between the intervention and control group at posttest (details in Additional file 12).

### Sensitivity analyses

The primary ITT models, testing for the effect of the intervention on CRF, cognitive and mental health outcomes, were additionally adjusted for *where* (home or school) and *when* (summer, holidays or autumn) participants completed the assessments. Adjusting for these confounding variables did not change the conclusions (i.e. all *p* > 0.05 or 95%CI included zero), but minor changes in the size and directionality of treatment effects were observed (details in Additional file 8).

We additionally repeated the primary ITT (Additional file 8), subgroup (Additional file 9), change-change (additional file 10), CACE (Additional file 11) and additional analysis (Additional file 12) on complete-case data. These analyses did not change the conclusions of the primary analyses (all *p* > 0.05).

## Discussion

In a large cohort of British adolescents, we assessed the effect of the Fit to Study scalable HIIT-style VPA intervention on secondary outcome measures including CRF, cognitive performance, and mental health. In line with findings for the primary outcome (attainment in maths [53]) but in contrast to our hypotheses, the HIIT-style VPA programme, incorporated into regular PE lessons for one academic year, did not significantly improve CRF, cognitive performance (EF, relational memory and processing speed), or mental health at posttest. Moreover, the effect of the intervention was not significantly moderated by sex, socioeconomic status or baseline CRF levels. Finally, one-year changes in CRF were not significantly related to changes in cognitive or mental health outcomes. Crucially, however, only a small proportion of schools complied with the intervention.

### Findings in context

Previous studies that examined the effect of HIIT and VPA interventions have shown robust effects on CRF in adolescents [34, 35, 76]. There is less research examining the effects of such interventions on cognitive- and mental health, but studies to date have shown improvements in EF [44–47, 77] and psychological health [45, 50], though a recent large-scale study found no support for these effects [37]. Despite the increasing body of evidence indicating the effectiveness of high-intensity exercise to improve health outcomes, we did not observe significant improvements in any of the outcome measures. Our findings that sex, socioeconomic status and baseline CRF did not significantly moderate the effects of the intervention on outcomes contrast with evidence from various meta-analyses showing that males [78] or females [79] and those with lower baseline CRF, may benefit most from high intensity PA interventions [80].

It has been hypothesised that changes in CRF – reflective of physiological changes – may mediate PA-related effects on cognitive- and mental health outcomes [81, 82]. In our study, both groups improved their CRF from pre to post-intervention. However, no group mean difference in CRF was found and, in line with findings from a recent meta-analysis [83], no significant relationships between changes in CRF and changes in cognitive performance or mental health were observed.

Given that existing evidence supports the positive impact of HIIT and VPA on CRF and suggests associated improvements in cognitive function and psychological health, it is important to interpret Fit to Study’s null results in the context of the trial: the absence of evidence is not evidence of absence [84]. Crucially, the Fit to Study intervention was limited by poor intervention fidelity, a common issue in the field of PA research [85], and provides an alternative explanation for the lack of the trial’s effectiveness. Fewer than half the schools in the intervention group managed to deliver the intervention in at least 50% of PE lessons during the year. Objective measures of VPA collected during single PE lessons demonstrated that the levels of VPA achieved were lower than the target level: approximately 4–5 min/hour of the active lesson was spent in VPA. Although we detected a trend towards higher VPA in the intervention schools, this failed to reach significance. Clearly, without a significant change or difference in exposure level (i.e. PA), no changes or differences in outcomes are to be expected. To explore the effect of compliance, we estimated the treatment effect in the population of compliers, which consisted of schools delivering the intervention in > 50% of lessons. These analyses did not change the results of the primary intention-to-treat analysis, although it is possible that a dose greater than 50% is required to observe a complier effect.

One of the main criticisms of HIIT-type interventions is the risk of poor adherence and attrition [39, 86], most likely due to the high intensity (and perceived exertion) inherent to HIIT. However, post-intervention teacher surveys, reported previously [53], suggested that not the intensity, but time constraints, lesson disruption (i.e. flow and objectives), lack of space (in combination with class size), seasonal variation and declining engagement due to lack of perceived improvements and repetitiveness of intervention elements, were important determinants of poor adherence in Fit to Study. Indeed, a recent meta-review reported mean adherence rates to be over 80% for HIIT interventions [58]. It is important to note, however, that fidelity is often poorly reported or not reported at all [58, 87], with possible overestimation of adherence due to publication bias. Despite the implementation challenges, schools reported that they would recommend the Fit to Study intervention to promote PA [53].

Moreover, although Fit to Study was evidence-based, its design prioritised the intervention being brief, inexpensive, simple, feasible and scalable. The intervention was therefore implemented in regular PE lessons, delivered by PE teachers, and adapted to suit the school PE curriculum, consisting of a potential total weekly dose of 20 min of HIIT-style VPA. The target duration and/or intensity was therefore low compared to the majority of interventions that have shown HIIT-related improvements in CRF, cognitive-, and mental health. In particular, cognitive- and mental improvements have been observed following 3–5 HIIT sessions per week [44–46, 50], while increased CRF levels have been reported following interventions delivered at higher intensities (> 85% max heart rate) [34]. Moreover, unlike previous studies, the Fit to Study HIIT-style infusions may have been interspersed with substantial (i.e. > 30 s - 3 min) recovery periods. Indeed, recent reviews suggest that interventions with intensities > 85% of the maximum heart rate, two-to-three times per week, with longer high intensity intervals (approx. 4 min) and active recovery periods (of approx. 3 min), lasting more than 7 weeks may provide the optimal stimulus for health improvement [36, 58, 80, 88]. While the Fit to Study intervention was delivered for 10 months, it may be that the actual dose of HIIT was too low or fragmented to observe clear effects, though low intervention compliance renders strong statements about dose tentative.

### Practical challenges of school-based PA interventions

Large-scale cluster RCTs embedded in school settings, like Fit to Study, have high ecological validity, but face many practical challenges that reduce the methodological quality of the trial and may introduce bias (see e.g. [32, 89] for reviews). Thorough implementation evaluations of Fit to Study [53], as well as a reflection paper [59], including recommendations for future HIIT-based PA trials, have been published elsewhere [53, 59] and various reviews have been published on school-related barriers [32, 33]; here we highlight challenges related to the scale and intervention approach of Fit to Study.

The scale of the Fit to Study trial was required to provide the statistical power to detect small treatment effects (in the primary outcome) and allowed for a representative sample of young adolescents, yet resulted in less control over the intervention (e.g. training, implementation) and poorer measurement of implementation fidelity. It was not possible for all PE teachers to attend the teacher training sessions, primarily due to time constraints. While teacher training videos were provided to help cascade instructions to colleagues, this process may have caused variability in teacher engagement and the quality of intervention delivery within and across schools. To measure such variability in implementation fidelity, objective and self-report measures were put in place. However, self-report measures are subject to response bias (i.e. social desirability [87, 90]), whereas objective measures of adherence, such as actigraphy or heart rate monitors, have major logistical and financial limitations when used at scale [91]. Smartphone-based applications may prove useful for fidelity measurement in future trials [92, 93].

The Fit to Study trial was designed to examine the efficacy of PA to improve maths attainment (secondarily: CRF, cognitive and mental health), and hence, whether HIIT-style VPA is a viable public health strategy for cognitive enhancement. As a consequence, we used a population-based approach [39, 89], rather than a targeted (e.g. sex-specific) approach, and designed the intervention and its outcomes to be scalable and feasible such that all pupils could participate. Such an inclusive approach prevents isolation and stigmatization [94], yet it is unlikely an intervention works for all, despite promising findings from feasibility testing in a small number of schools (unpublished). Indeed, there is evidence that girls may respond better to PA interventions than boys [95], whereas high intensity PA may show greater cognitive benefits in boys [78]. Similarly, the intervention was easily incorporated into lesson plans of some schools, but proved incompatible with lesson objectives and timetables in others [53]. Schools that struggled with delivering all intervention elements were allowed to omit the third infusion occasionally [53], in order to keep schools, teachers and pupils engaged. Such modifications are not desirable, but suggest that flexibility in intervention delivery may be required [51]. Lack of time was the major barrier [53], not only for implementation of the intervention, but also for conducting secondary outcome assessments. Variability in IT-facilities further complicated completion of assessments, resulting in great amounts of missing data. Future trials are encouraged to use implementation frameworks in the design phase to guide implementation and scale-up of PA trials [51].

### Strengths and limitations

Strengths of the study include its novelty in implementing a HIIT-style intervention at scale in a real-world setting, it being a highly scalable intervention, which was delivered at low cost [53], its broad range of secondary outcome measures (which we have already shown to be sensitive to other effects, e.g. between fitness and mental health at baseline [96]), its statistical techniques to deal with missing data, and its large cohort of young adolescents, who reported baseline PA levels that were representative of the UK [97] and the world [21], ensuring generalisability.

The study also has various limitations in addition to poor intervention fidelity and large amounts of missing data. Although we collected self-report data on compliance with the intervention, we did not measure whether individuals reached the required intensity because distribution of heart rate monitors to all participants was unfeasible. It is paramount that future studies put in place measures that capture the multidimensional nature of fidelity [98, 99]. No active control group was used in this efficacy trial, and hence, no conclusions regarding the optimal dose can be drawn. Finally, there is some evidence that HIIT interventions may have greater benefits in overweight / obese children and adolescents [37] and in pubertal children compared to pre-pubertal children [36], yet due to the scale of the trial, the use of opt-out consent, and sensitive nature of pubertal questionnaires, no information on weight or pubertal status was collected.

## Conclusion

The one-academic-year HIIT-style VPA intervention had no significant effect on CRF, cognitive performance, or mental health in young adolescents. Large amounts of missing data and poor fidelity with the intervention limit the extent to which conclusions can be drawn regarding the causal relationship of physical activity and health outcomes in adolescents. Future well-controlled trials testing for the effects of HIIT-style interventions are warranted.

## Supplementary Information

**Additional file 1:.** CONSORT checklist

**Additional file 2:.** TIDieR checklist

**Additional file 3:.** Supplementary methods

**Additional file 4:.** Missing data overview

**Additional file 5:.** Comparison of school drop-outs pre-baseline assessments

**Additional file 6:.** Data cleaning

**Additional file 7:.** Baseline school, PA and PE characteristics

**Additional file 8:.** Additional information for primary outcome analyses

**Additional file 9:.** Additional information for subgroup analyses

**Additional file 10:.** Additional information for change-change analyses

**Additional file 11:.** Intervention fidelity and complier-average causal effect (CACE) analysis

**Additional file 12:.** Additional analyses of PE enjoyment and attitudes towards PA

## Data Availability

The datasets generated and analysed during the current study are not publicly available due to the sensitivity of the data, but are available from the corresponding author on reasonable request.

## Abbreviations

20MSR: 20-m shuttle run
CACE: Complier-average causal effect
CONSORT: Consolidated Standards of Reporting Trial guidelines
CRF: Cardiorespiratory fitness
EF: Executive functions
eFSM: Eligible for free school meals
HIIT: High-intensity interval training
ITT: Intention-to-treat
MAR: Missing at random
MICE: Multiple imputation by chained equations
MVPA: Moderate-to-vigorous physical activity
NFER: National Foundation for Educational Research
PA: Physical activity
PE: Physical education
RCT: Randomised controlled trial
SDQ: Strength and Difficulties Questionnaire
SEM: Structural equation model
SMD: Standardized mean difference
TIDieR: Template for intervention description and replication
VPA: Vigorous physical activity
