# Supplementary material for: The effect of a one-year vigorous physical activity intervention on fitness, cognitive performance and mental health in young adolescents: the Fit to Study cluster randomised controlled trial
Source: Int J Behav Nutr Phys Act. 2021 Mar 31;18:47. doi: 10.1186/s12966-021-01113-y (PMC8011147; doi:10.1186/s12966-021-01113-y)
Supplement: Supplementary file 3 — Additional file 3:. Supplementary methods [file 12966_2021_1113_MOESM3_ESM.docx]

**Additional File 3. Supplementary methods**

**Physical activity measures**

Self-reported PA was used to characterise baseline PA levels of the sample. PA in the past week was assessed using a single item (1) asking: “In the past week, on how many days have you done a total of 60 minutes or more of physical activity, which was enough to raise your breathing rate? This may include sport, exercise, and brisk walking or cycling for fun, or to get to and from places.” (range 0 - 7). Habitual PA over the past six months was measured with a single item drawn from previous studies (2): “Thinking about the past six months, how often have you been physically active for an hour every day during a typical week in school term?" (1 = never to 7 = always).

**Cognitive assessments**

*Reaction time task*

During the task, participants were instructed to respond as quickly as possible (pressing the spacebar) to the presentation of a triangle on the screen (40 trials). Responses between 100-3,000 ms were considered valid.

*Relational memory task*.

In the task, participants were presented with two blocks of 18 study trials (i.e. events) containing scene, face and object stimuli. Within each trial, each type of stimulus was presented individually and sequentially (1,300 ms). Participants were instructed to memorise the stimuli within each trial as belonging to a single event. During the recognition phase, participants were shown 36 test trials, each containing a place, object and face stimulus, half of which were intact (i.e. the stimuli appeared in the same study trial). Participants were instructed to indicate whether all stimuli were from the same even, or whether one of the stimuli did not belong to the event. Responses within 200-15,000 ms were considered valid.

*Modified flanker task*

Participants were instructed to respond as quickly and accurately as possible to the direction of a centrally presented target arrow, which was flanked by arrows that were either in the same direction (i.e. congruent) or opposite direction (i.e. incongruent) to the centrally presented target arrow. The task consisted of two blocks of 52 trials each and responses within 200-1,400 ms were considered valid. Accuracy and average reaction times on congruent and incongruent valid trials were recorded.

*Visual two-back*

Participants were shown pseudorandom sequences of weather symbols (i.e. sun, moon, rainbow, snowflake and star), and instructed to identify as quickly and accurately as possible whether the stimulus on the current trial was identical to the one presented two trials ago. The task consisted of two blocks of 26 trials each and responses within 150-2,500 ms were considered valid. The accuracy and average reaction time on valid trials were recorded.

*Colour-shape switching task*

Participants were instructed to learn a set of response actions that were related to the colour or shape of a centrally presented character: press the “F” key if the character was blue or a circle and the “J” key if the character was green or square. The direction of the character’s arms indicated whether to pay attention to its shape or colour. During homogeneous blocks (2 blocks of 32 trials), participants had to pay attention to either the colour or the shape. During heterogeneous blocks (2 blocks of 64 trials), participants had to flexibly shift their attention towards either the character’s shape or colour based on the direction of the character’s arms. The direction of the character’s arms switched every two trials, giving rise to switch trials and non-switch trials. Responses within 200-6,000 ms were considered valid. Accuracy and average reaction times on switch and non-switch trials were recorded.

**Actigraphy during PE**

Objective measures of PA during PE were collected using wrist-worn AX3 tri-axial accelerometers (Open Lab, University of Newcastle, UK). Participants wore the accelerometer on their non-dominant wrist. Accelerometers were set to record at 100 Hz and were attached after participants changed for PE and collected before they changed back into school clothes. Following conversion of raw accelerometer data into 1 s epochs, class average minutes of sedentary PA (SPA), moderate PA (MPA) and vigorous PA (VPA) during lesson time were calculated using established cut-points (3) and standardised to minutes per hour. We computed the full lesson PA estimates as well as the active, or “effective”, PA estimates. The former represents PA estimates standardized to total time allocated for PE and the latter represent the amount of PA adjusted for change time.

**Data cleaning**

*Cognitive assessments*

Cognitive data were cleaned per assessment. For each assessment, we identified participants with fewer than 30% valid trials (i.e. out of the total number of trials), under the assumption that a proportion (>30%) of trials is required to achieve a reliable estimate of each cognitive construct. Furthermore, for the relational memory and visual two-back task, we identified participants who consistently pressed a single response button throughout the task, as indicated by zero counts for false alarms and hits, or by zero counts for misses and correct rejections. We additionally inspected the primary outcome measures of each task and removed participants with values that were outside the expected range (i.e. 0-100 for accuracy outcomes, and mean RT outside the pre-defined RT bounds, see *cognitive performance* section above) or were missing due to data collection errors.

*Questionnaire assessments*

Questionnaire measures included in this study were part of a larger battery that consisted of three blocks Likert-scale based items. Long strings of identical answers (i.e. same position on the scale) within a block, accounting for reversed items, were identified. The mean string length (MSL) per block was computed, and data were removed if participants gave the same answer throughout a block and recorded a string of MSL+/- 3SD in at least one more block. In addition, we removed participant scores that were outside the range of individual items (e.g. score > 5 on 5-point Likert scale) due to data collection errors.

*Fitness assessments*

Schools that completed the 12min Cooper Run test (n = 4, of which 1 intervention school; 561 pupils) were excluded from the analysis. Due to the difference in scale and units of the outcome measures (20MSR: laps versus Cooper: total distance), the scores of these tests cannot easily be combined. Classically, researchers have used estimating equations to convert 20MSR (4–9) and Cooper Test performance(10,11) to estimated VO_2max_ values. While the conversion to a common metric is attractive, we decided not to pursue this because: (1) a large number of estimating equations exist, particularly for 20MSR performance (>17) but selecting one that is appropriate for the Fit to Study sample is not straightforward. Depending on the fitness protocol used, these equations vary in their predictors, most commonly including one or more of sex, age, speed or body weight, and more generally in their accuracy of predicting VO_2max_ (5,6). Second, estimating equations may underestimate (11,12) or overestimate (13) an individual's real VO_2max._ This is particularly problematic if the degree of under / over estimation differs between the Cooper test and 20MSR. A comparison of estimated VO_2max_ values from 20MSR and the Cooper test in healthy adults demonstrated that, although 20MSR underestimated an individual's level of cardiorespiratory fitness, the converted Cooper test results were biased: underestimation of VO_2max_ values in low fit individuals and overestimation of VO_2max_ in high fit individuals (12). Therefore, the 20MSR results were treated as the main measure of cardiorespiratory fitness.

**Minimum detectable effect size**

Sample size calculations were performed by NatCen for the primary intention-to-treat analysis, which compared maths test performance between intervention and control schools (14). The initial sample size calculation demonstrated a range of minimum detectable effect sizes (MDES) varying between 0.18-0.26 for various achieved sample sizes (80-100 schools), assuming 80% power, alpha=0.05 (two-tailed), 90 pupils per school and baseline covariates explaining 50% of variance at pupil and school level. These estimates were updated (in February- March 2018) to reflect school drop-out (n = 18 schools) and a revised set of assumptions (15). In particular, the new MDES were computed based on: 88 participating schools (47% intervention), on average 78 pupils per school, baseline covariates explaining 40% of pupil level variance and 10% of school level variance and an ICC of 0.16. The newly computed MDES was equal to 0.24 standard deviations. The MDES would have been 0.21 standard deviations if all 106 recruited schools had participated in the trial. At the final analysis stage, the MDES was calculated to be 0.22 SD (16). Power calculations were performed in PowerUp! (17). No *a priori* sample size calculations were performed for the secondary outcomes, but an indication of the MDES is provided below.

The MDES for the cognitive tests was calculated for the randomisation sample using the following set of assumptions: (1) 104 schools, 50% of which in the intervention group, (2) an average of 176 pupils per school (N = 18,261), (3) baseline covariates explaining 40% of pupil level variance and 10% of school level variance (based on NatCen's adjusted estimates), (4) an ICC of 0.02, following a previous study measuring executive functions in a comparable sample (18) and (5) 80% power with alpha=0.05 (two-tailed). Under these assumptions MDES was 0.07 SD (95% CI: 0.027-0.16; it would be 0.15 SD, 95% CI: 0.045-0.12, if the ICC was 0.06 (19)). With a similar set of assumptions and an ICC = 0.25 (NB. teacher-rated; (20)), the MDES for the SDQ of the questionnaire was 0.23 SD. For cardiorespiratory fitness, the MDES was 0.08 SD with a similar set of assumptions and an ICC = 0.03 (21). Notably, the trial was not designed to measure or affect cognitive or mental health scores (being secondary measures), hence this MDES solely serves as an *a-posteriori* indication of a possible detectable effect size under this particular set of assumptions. The MDES for the secondary outcomes was not updated *a-posteriori*, given the differential amounts of missingness across outcome measures and the lack of intervention fidelity of the trial.

**CACE analysis**

The ITT analysis will provide a conservative (or underestimated) treatment effect when schools do not adhere to the intervention (22). We therefore estimated a complier average causal effect (CACE, or local average causal effect, LATE) to estimate the average treatment effect in the population of compliers. The CACE was estimated under one-sided non-compliance, by fitting an instrumental variable (IV) model using the two-stage least squares method. The IV model included the assigned treatment as the instrumental variable and adjusted for the stratification variable (school gender type) and baseline values of the respective outcome measure. In line with the analysis of the primary outcome (16), we used the compliance estimates of the post-intervention teacher-reported school survey as primary compliance metric. A total of 22 intervention schools (of 29 at posttest) provided compliance information. Intervention schools in which the intervention was delivered in > 50% of PE lessons were classified as compliant. Schools that did not provide compliance data were assumed to be non-compliant. The control group was included in the IV model with their treatment received set to 0.

**Missing data**

*Primary analysis*

Pupils with missing outcome data were more likely to be male, from a lower socioeconomic background (i.e. eFSM) and to have completed the baseline assessments at school (*p* < 0.05). Moreover, for each assessment, pupils with more favourable outcomes at baseline were less likely to have missing outcome data. School-level variables did not significantly predict school drop-out (*p* > 0.05).

The dataset was multiply imputed (m = 180 imputations, determined using a recently proposed two-stage procedure (23)) using multilevel multiple imputation by chained equations (MICE) in R. The imputation model included all variables that were part of the analysis model, as well as any variables that were associated (*r* > 0.4) with variables in those models or related with missingness in variables in the analysis model (24,25). Models that contained all cognitive assessment date variables (i.e. *when* assessments were conducted) at post-test resulted in errors. Given that (1) only 37 participants (of 6,174) completed a cognitive assessment during summer or autumn term at posttest, and (2) the reaction time task was always completed first, we therefore decided to only include the variable indicating when the reaction time task was completed. An overview of all variables included in the imputation model is provided in Additional File 4 (Table 3). Moreover, due to computational issues, we removed some variables from the final set of imputation models, reported in Additional file 4 (Table 4).

Continuous variables were imputed using a two-level normal model with homogeneous within group variances (PAN) and categorical variables using two-level predictive mean matching. The data was imputed separately by treatment group to allow group-specific covariance structures, thereby preserving all two-way interactions between the treatment group and other variables in the imputation model (26). The imputations were visually checked for convergence and estimates were pooled using Rubin’s rules (27).

*CACE analysis*

We separately imputed the data for the complier average causal effect (CACE) analysis, given the large amount of missingness in the fidelity measure. The imputation models were identical to the ones included in the primary multiple imputation analysis, with the addition of a compliance indicator. Intervention schools in which the intervention was delivered in > 50% of PE lessons were classified as compliant and all other schools were classified as non-compliant (i.e. not receiving the active intervention). We opted to multiply impute the data for the CACE analysis separately for several reasons: (1) the compliance measure that was used for the CACE analysis was missing for 24 out of 46 (i.e. 52%) intervention schools, that were assumed to be non-compliant with the intervention, and (2) the measure was deemed a proxy of compliance, given that it was reported by teachers following the intervention period, making it subject to various forms of bias (e.g. recall bias).

**Software**

The majority of the analyses were conducted in Rstudio (28) with R ((29), version 4.0) using the *estimatr* package (30). Plots were created using *ggplot2* (31)*.* Missing data was imputed using MICE (32) on the high-performance cluster of the Wellcome Centre for Integrative Neuroimaging, Oxford UK (R, version 3.6). The structural equation model was constructed using Mplus 8.4 (33).

**References**

1. Scott JJ, Morgan PJ, Plotnikoff RC, Lubans DR. Reliability and validity of a single‐item physical activity measure for adolescents. J Paediatr Child Health. 2015;51(8):787–93.

2. Hagger MS, Chatzisarantis N, Biddle SJH, Orbell S. Antecedents of children’s physical activity intentions and behaviour: Predictive validity and longitudinal effects. Psychol Heal. 2001;16(4):391–407.

3. Phillips LRS, Parfitt G, Rowlands A V. Calibration of the GENEA accelerometer for assessment of physical activity intensity in children. J Sci Med Sport. 2013;16(2):124–8.

4. Tomkinson GR, Lang JJ, Tremblay MS, Dale M, Leblanc AG, Belanger K, et al. International normative 20 m shuttle run values from 1142026 children and youth representing 50 countries. Br J Sports Med. 2017;51:1545–54.

5. Batista MB, Cyrino EdS, Arruda M, Dourado AC, Coelho-E-Silva MJ, Ohara D, et al. Validity of equations for estimating VO2peak from the 20-m shuttle run test in adolescents aged 11-13 years. J Strength Cond Res. 2013;27(10):2774–81.

6. Melo X, Santa-Clara H, Almeida JP, Carnero EA, Sardinha LB, Bruno PM, et al. Comparing several equations that predict peak VO2 using the 20-m multistage-shuttle run-test in 8-10-year-old children. Eur J Appl Physiol. 2011;111(5):839–49.

7. Matsuzaka A, Takahashi Y, Yamazoe M, Kumakura N, Ikeda A, Wilk B, et al. Validity of the multistage 20-m shuttle-run test for Japanese children, adolescents, and adults. Pediatr Exerc Sci. 2004;16(2):113–25.

8. Léger LA, Mercier D, Gadoury C, Lambert J. The multistage 20 metre shuttle run test for aerobic fitness. J Sports Sci. 1988;6(2):93–101.

9. Mahar MT, Welk GJ, Rowe DA. Estimation of aerobic fitness from PACER performance with and without body mass index. Meas Phys Educ Exerc Sci. 2018;22(3):239–49.

10. Cooper KH. A Means of Assessing Maximal Oxygen Intake. JAMA. 1968;203(3):201–4.

11. Grant S, Corbett K, Amjad AM, Wilson J, Aitchison T. A comparison of methods of predicting maximum oxygen uptake. Br J Sports Med. 1995;29(3):147–52.

12. Penry JT, Wilcox AR, Yun J. Validity and reliability analysis of Cooper’s 12-minute run and the multistage shuttle run in healthy adults. J Strength Cond Res. 2011;25(3):597–605.

13. Scott SN, Springer CM, Oody JF, McClanahan MS, Wiseman BD, Kybartas TJ, et al. Development and validation of a PACER prediction equation for VO2peak in 10-to 15-year-old youth. Pediatr Exerc Sci. 2019;31(2):223–8.

14. Husain F. Fit to Study. Evaluation protocol [Internet]. 2016. Available from: https://educationendowmentfoundation.org.uk/projects-and-evaluation/projects/fit-to-study/

15. Bartasevicius V, Jabin N. Statistical Analysis Plan for the efficacy trial of the Fit to Study intervention [Internet]. 2018. Available from: https://educationendowmentfoundation.org.uk/public/files/Projects/Fit_to_Study_SAP_2018.03.15_FINAL.pdf

16. Husain F, Bartasevicius V, Marshall L, Chidley S, Forsyth E. Fit to study. Evaluation report. [Internet]. 2019. Available from: https://educationendowmentfoundation.org.uk/public/files/FitToStudy.pdf

17. Dong N, Maynard R. PowerUp!: A Tool for Calculating Minimum Detectable Effect Sizes and Minimum Required Sample Sizes for Experimental and Quasi-Experimental Design Studies. J Res Educ Eff. 2013;6(1):24–67.

18. Aadland KN, Ommundsen Y, Anderssen SA, Brønnick KS, Moe VF, Resaland GK, et al. Effects of the Active Smarter Kids (ASK) Physical Activity School-based Intervention on Executive Functions: A Cluster-Randomized Controlled Trial. Scand J Educ Res. 2019;63(2):214–28.

19. Aadland KN, Moe VF, Aadland E, Anderssen SA, Resaland GK, Ommundsen Y. Relationships between physical activity, sedentary time, aerobic fitness, motor skills and executive function and academic performance in children. Ment Health Phys Act. 2017;12:10–8.

20. Berry V, Axford N, Blower S, Taylor RS, Edwards RT, Tobin K, et al. The Effectiveness and Micro-costing Analysis of a Universal, School-Based, Social–Emotional Learning Programme in the UK: A Cluster-Randomised Controlled Trial. School Ment Health. 2016;8(2):238–56.

21. Kriemler S, Zahner L, Schindler C, Meyer U, Hartmann T, Hebestreit H, et al. Effect of school based physical activity programme (KISS) on fitness and adiposity in primary schoolchildren: cluster randomised controlled trial. BMJ. 2010;340:c785.

22. Hernán MA, Hernández-Díaz S. Beyond the intention-to-treat in comparative effectiveness research. Clin Trials. 2012;9(1):48–55.

23. Von Hippel PT. How Many Imputations Do You Need? A Two-stage Calculation Using a Quadratic Rule. Sociol Methods Res. 2020;49(3):699–718.

24. Van Buuren S. Flexible imputation of missing data. Chapman and Hall/CRC; 2018.

25. Van Buuren S, Boshuizen HC, Knook DL. Multiple imputation of missing blood pressure covariates in survival analysis. Stat Med. 1999;18:681–94.

26. Enders CK, Gottschall AC. Multiple imputation strategies for multiple group structural equation models. Struct Equ Model. 2011;18(1):35–54.

27. Rubin DB. Multiple Imputation for NonResponse in Surveys. New-York: Wiley; 1987.

28. RStudio Team. RStudio: Integrated Development Environment for R [Internet]. Boston, MA; 2016. Available from: http://www.rstudio.com/

29. R Core Team. R: A Language and Environment for Statistical Computing [Internet]. Vienna, Austria; 2020. Available from: https://www.r-project.org/

30. Blair G, Cooper J, Coppock A, Humphreys M, Sonnet L. estimatr: Fast Estimators for Design-Based Inference [Internet]. 2020. Available from: https://cran.r-project.org/package=estimatr

31. Wickham H. ggplot2: Elegant Graphics for Data Analysis [Internet]. 2016. Available from: https://cran.r-project.org/package=ggplot2

32. Van Buuren S, Groothuis-oudshoorn K. MICE: Multivariate Imputation by Chained Equations in R. J Stat Softw. 2011;45(3):1–67.

33. Muthén LK, Muthén BO. Mplus User’s Guide. Eighth Edition. Los Angeles, CA, USA: Muthén & Muthén;
