# Supplementary material for: The effect of a one-year vigorous physical activity intervention on fitness, cognitive performance and mental health in young adolescents: the Fit to Study cluster randomised controlled trial
Source: Int J Behav Nutr Phys Act. 2021 Mar 31;18:47. doi: 10.1186/s12966-021-01113-y (PMC8011147; doi:10.1186/s12966-021-01113-y)
Supplement: Supplementary file 4 — Additional file 4:. Missing data overview [file 12966_2021_1113_MOESM4_ESM.docx]

**Additional file 4. Missing data**

Tables 1 and 2 contain the number (% of total) of missing cases of, respectively, school- and pupil-level variables that were used in the analyses (arranged by increasing proportion of missingness). There was no missing data within each cognitive assessment, hence a single variable is used to represent each cognitive assessment. The proportion of missing values is > 70% for all cognitive assessments but the reaction time task at posttest.

Only 630 participants completed all assessments (CRF, questionnaire and cognitive tasks) at both timepoints (~4%). No information was collected on pupil-level drop-out.

**Table 1. Missing data (proportion) of school-level variables considered in this study**

|  | No missing | % missing (of 93) |
| --- | --- | --- |
| Gender status | 0 | 0 |
| School type | 0 | 0 |
| Establishment type | 0 | 0 |
| School size | 0 | 0 |
| IMD decile | 0 | 0 |
| Proportion of eFSM pupils | 0 | 0 |
| Teacher training received | 0 | 0 |
| Ofsted rating | 1 | 1.08 |
| Number of Year 7 form groups | 2 | 2.15 |
| School level averaged actigraphy pretest | 3 | 3.23 |
| School level averaged actigraphy posttest | 22 | 23.66 |
| Teacher-reported fidelity | 71 | 76.34 |

Abbreviations: IMD = index of multiple deprivation, eFSM = eligible for free school meals

**Table 2. Missing data (proportion) of pupil-level variables considered in this study (arranged by percentage of missingness)**

|  | **No missing** | **% missing** |
| --- | --- | --- |
| age | 0 | 0 |
| sex | 0 | 0 |
| eFSM | 0 | 0 |
| 20MSR term posttest | 0 | 0 |
| 20MSR term pretest | 0 | 0 |
| 20MSR pretest | 5320 | 33.21 |
| PE enjoyment pretest | 6243 | 38.98 |
| Internalising score pretest | 6245 | 38.99 |
| Externalising score pretest | 6246 | 39 |
| Questionnaire date pretest | 6272 | 39.16 |
| Questionnaire location pretest | 6272 | 39.16 |
| Physical self-esteem pretest | 6296 | 39.31 |
| Global self-esteem pretest | 6297 | 39.31 |
| Self-reported PA pretest | 6318 | 39.45 |
| 20MSR posttest | 7903 | 49.34 |
| Reaction time task pretest | 8077 | 50.43 |
| Pupil-reported fidelity: warm-up | 9603 | 59.96 |
| Pupil-reported fidelity: infusions | 9603 | 59.96 |
| Pupil-reported fidelity: participation | 9603 | 59.96 |
| Internalising score posttest | 9605 | 59.97 |
| Externalising score posttest | 9609 | 59.99 |
| Self-reported PA posttest | 9619 | 60.05 |
| Questionnaire date posttest | 9623 | 60.08 |
| Questionnaire location posttest | 9623 | 60.08 |
| Global self-esteem posttest | 9868 | 61.61 |
| Physical self-esteem posttest | 9868 | 61.61 |
| Reaction time task posttest | 9878 | 61.67 |
| Flanker task pretest | 10569 | 65.99 |
| Relational memory task pretest | 10699 | 66.8 |
| Two-back pretest | 11005 | 68.71 |
| Colour-shape task switch pretest | 11295 | 70.52 |
| Flanker task posttest | 11500 | 71.8 |
| Two-back posttest | 11516 | 71.9 |
| Relational memory task posttest | 11525 | 71.95 |
| Teacher-reported fidelity | 11963 | 74.69 |
| Colour-shape task switch posttest | 12081 | 75.43 |

Abbreviations: 20MSR = 20 meter shuttle run, eFSM = eligible for free school meals, PA = physical activity

*Missing data patterns*

A total of 1,590 unique missing data patterns were present in the data. To provide an overview of some of the patterns, we created an intersection plot (using UpsetR in R (1)) displaying the patterns of complete data for those subjects who completed the assessments at both timepoints (Figure 1).

**Figure 1. Intersection plot demonstrating patterns of completion of assessments (pre and posttest).** *Left bar plot*: This bar plot contains the total number of cases who completed each assessment, the name of which is displayed at the right side of the barplot**.** *Middle circle plot*: This plot shows the intersections, i.e. whether a participant completed one or more assessments. A closed dark blue circle denotes that a participant completed the task and vertical lines connecting circles refer to the set of assessments that were completed**.** *Top bar plot*: This bar plot shows the frequency of each intersection, i.e. the total number of participants who completed one or more assessments, as indicated by the circle plot below. For instance, the left most bar in the top barplot indicates that over 2,500 cases had data for the fitness assessment (pre and post) only.

***Variables included in multiple imputations***

The imputation model included all variables that were part of the analysis model, as well as any variables that were associated (*r* > 0.4) with variables in those models or related with missingness in variables in the analysis models. Models that contained all cognitive assessment date variables (i.e. *when* assessments were conducted) at post-test resulted in errors. Given that (1) only 37 participants (of 6,174) completed a cognitive assessment during summer or autumn term at posttest, and (2) the reaction time task was always completed first, we therefore decided to only include the variable indicating when the reaction time task was completed. An overview of all variables included in the imputation model is provided in Table 3. Moreover, due to computational issues, we removed some variables from the final set of imputation models, reported in Table 4.

**Table 3.** **Variables included in the multiple imputations**

| # | **Variables** |
| --- | --- |
| 1 | School |
| 2 | School gender type |
| 3 | School percentage eFSM upils |
| 4 | school size |
| 5 | age |
| 6 | sex |
| 7 | eFSM |
| 8 | Flanker RT congruent (tp1) |
| 9 | Flanker RT congruent (tp2) |
| 10 | Flanker RT incongruent (tp1) |
| 11 | Flanker RT incongruent (tp2) |
| 12 | Flanker date / term (tp1) |
| 13 | Flanker location (tp1) |
| 14 | Flanker location (tp2) |
| 15 | Flanker accuracy incongruent (tp1) |
| 16 | Flanker accuracy incongruent (tp2) |
| 17 | Flanker accuracy congruent (tp1) |
| 18 | Flanker accuracy congruent (tp2) |
| 19 | Two-back accuracy (tp1) |
| 20 | Two-back accuracy (tp2) |
| 21 | Two-back RT (tp1) |
| 22 | Two-back RT (tp2) |
| 23 | Two-back date / term (tp1) |
| 24 | Two-back location (tp1) |
| 25 | Two-back location (tp2) |
| 26 | Reaction time task RT (tp1) |
| 27 | Reaction time task date / term (tp1) |
| 28 | Reaction time task (tp1) |
| 29 | Reaction time task RT (tp2) |
| 30 | Reaction time task date / term (tp2) |
| 31 | Reaction time task (tp2) |
| 32 | Task switch RT switch (tp1) |
| 33 | Task switch RT switch (tp2) |
| 34 | Task switch RT non-switch (tp1) |
| 35 | Task switch RT non-switch (tp2) |
| 36 | Task switch date / term (tp1) |
| 37 | Task switch location (tp1) |
| 38 | Task switch location (tp2) |
| 39 | Task switch accuracy switch (tp1) |
| 40 | Task switch accuracy non-switch (tp1) |
| 41 | Task switch accuracy switch (tp2) |
| 42 | Task switch accuracy non-switch (tp2) |
| 43 | Relational memory accuracy (tp1) |
| 44 | Relational memory accuracy (tp2) |
| 45 | Relational memory RT (tp1) |
| 46 | Relational memory RT (tp2) |
| 47 | Relational memory date / term (tp1) |
| 48 | Relational memory location (tp1) |
| 49 | Relational memory location (tp2) |
| 50 | Q internalising (tp1) |
| 51 | Q internalising (tp2) |
| 52 | Q externalising (tp1) |
| 53 | Q externalising (tp2) |
| 54 | Q global self esteem (tp1) |
| 55 | Q physical self esteem (tp1) |
| 56 | Q global self esteem (tp2) |
| 57 | Q physical self esteem (tp2) |
| 58 | Q location (tp1) |
| 59 | Q location (tp2) |
| 60 | 20MSR term (tp1) |
| 61 | 20MSR term (tp2) |
| 62 | 20MSR (tp1) |
| 63 | 20MSR (tp2) |
| 64 | Q date / term (tp1) |
| 65 | Q date / term (tp2) |
| 66 | Q PA past week (tp1) |
| 67 | Q PA past week (tp2) |
| 68 | Q attitude (tp1) |
| 69 | Q attitude (tp2) |
| 70 | Q enjoy PE (tp1) |
| 71 | Q enjoy PE (tp2) |
| 72 | Q habitual PA (tp1) |
| 73 | Q habitual PA (tp2) |
| 74 | Q willingness (tp1) |
| 75 | Q willingness (tp2) |
| 76 | Q self-health (tp1) |
| 77 | Q self-health (tp2) |
| 78 | Q similar to active (tp1) |
| 79 | Q similar to active (tp2) |
| 80 | Q intent to be active (tp1) |
| 81 | Q intent to be active (tp2) |
| 82 | School level fidelity |

Abbreviations: 20MSR = 20-meter shuttle run, eFSM = eligible for free school meals, Q = questionnaire, RT = reaction time, tp = timepoint

**Table 4**. **Excluded variables from imputation models**

| **Variable name** | **Excluded from** |
| --- | --- |
| Reaction time task date / term (tp2) | Imputation models for baseline (tp1) Flanker or task-switching performance |
| Q date / term (tp1) | Imputation models of task-switching (tp2) scores |
| 20MSR term (tp1)  20MSR term (tp2) | Only used to impute 20MSR scores |

Abbreviations: 20MSR = 20-meter shuttle run, tp = timepoint

**References**

1. Conway JR, Lex A, Gehlenborg N. UpSetR: An R package for the visualization of intersecting sets and their properties. Bioinformatics. 2017;33(18):2938–40.
