# Supplementary material for: The effect of a one-year vigorous physical activity intervention on fitness, cognitive performance and mental health in young adolescents: the Fit to Study cluster randomised controlled trial
Source: Int J Behav Nutr Phys Act. 2021 Mar 31;18:47. doi: 10.1186/s12966-021-01113-y (PMC8011147; doi:10.1186/s12966-021-01113-y)
Supplement: Supplementary file 5 — Additional file 5:. Comparison of school drop-outs pre-baseline assessments [file 12966_2021_1113_MOESM5_ESM.docx]

**Additional file 5. Drop-out of schools at pre-baseline assessments**

**School drop-out pre-baseline**

Eleven schools (6 intervention, 5 control) dropped-out post-randomisation but prior to baseline assessments, because of time constraints (n = 3), staff changes (n = 2), inability to commit (n = 1) or unknown reasons (n = 4). These schools were unaware of their treatment allocation at the time of drop-out. Table 1 presents characteristics of schools and pupils that remained in the trial and those that dropped out of the trial.

A comparison between schools that dropped-out and remained part of the trial showed no significant differences in any of the school-level or pupil level demographics (all *p* > 0.05). In particular, a bias-reduced generalised linear model (1,2) was fit to examine whether any of the school level variables predicted drop-out. The analysis of deviance table (type II Wald chi-square tests) is provided in Table 2. We then fit a multilevel logistic regression (with logit link) to examine whether any pupil level characteristics predicted drop-out (Table 3). The remaining 93 schools were available for baseline assessments and are considered the main trial baseline sample.

**Table 1. School level and pupil level characteristics of drop-outs and those that remained part of the trial**

|  | **Control** | | **Intervention** | | **Overall** | |
| --- | --- | --- | --- | --- | --- | --- |
|  | **Included** | **Drop-out** | **Included** | **Drop-out** | **Included** | **Drop-out** |
| **SCHOOL LEVEL** |  |  |  |  |  |  |
| No. schools | 47 | 5 | 46 | 6 | 93 | 11 |
| **Gender status** |  |  |  |  |  |  |
| Co-ed | 38 (80.9%) | 4 (80.0%) | 37 (80.4%) | 5 (83.3%) | 75 (80.6%) | 9 (81.8%) |
| Female | 8 (17.0%) | 0 (0%) | 9 (19.6%) | 1 (16.7%) | 17 (18.3%) | 1 (9.1%) |
| Male | 1 (2.1%) | 1 (20.0%) | 0 (0%) | 0 (0%) | 1 (1.1%) | 1 (9.1%) |
| **Establishment type** |  |  |  |  |  |  |
| Academy - Sponsor Led | 9 (19.1%) | 0 (0%) | 7 (15.2%) | 2 (33.3%) | 16 (17.2%) | 2 (18.2%) |
| Academy Converter | 1 (2.1%) | 0 (0%) | 2 (4.3%) | 0 (0%) | 3 (3.2%) | 0 (0%) |
| Academy Converter - Mainstream | 15 (31.9%) | 3 (60.0%) | 16 (34.8%) | 1 (16.7%) | 31 (33.3%) | 4 (36.4%) |
| Community School | 7 (14.9%) | 1 (20.0%) | 8 (17.4%) | 2 (33.3%) | 15 (16.1%) | 3 (27.3%) |
| Foundation School | 5 (10.6%) | 1 (20.0%) | 5 (10.9%) | 0 (0%) | 10 (10.8%) | 1 (9.1%) |
| Free School - Mainstream | 2 (4.3%) | 0 (0%) | 3 (6.5%) | 0 (0%) | 5 (5.4%) | 0 (0%) |
| Voluntary aided school | 7 (14.9%) | 0 (0%) | 4 (8.7%) | 1 (16.7%) | 11 (11.8%) | 1 (9.1%) |
| Voluntary controlled school | 1 (2.1%) | 0 (0%) | 1 (2.2%) | 0 (0%) | 2 (2.2%) | 0 (0%) |
| **School type** |  |  |  |  |  |  |
| Comprehensive to 16 | 9 (19.1%) | 2 (40.0%) | 7 (15.2%) | 3 (50.0%) | 16 (17.2%) | 5 (45.5%) |
| Comprehensive to 18 | 33 (70.2%) | 3 (60.0%) | 34 (73.9%) | 3 (50.0%) | 67 (72.0%) | 6 (54.5%) |
| Secondary Modern | 5 (10.6%) | 0 (0%) | 5 (10.9%) | 0 (0%) | 10 (10.8%) | 0 (0%) |
| **School size** |  |  |  |  |  |  |
| Large (> 200) | 13 (27.7%) | 1 (20.0%) | 14 (30.4%) | 4 (66.7%) | 27 (29.0%) | 5 (45.5%) |
| Medium (100-200) | 27 (57.4%) | 4 (80.0%) | 27 (58.7%) | 2 (33.3%) | 54 (58.1%) | 6 (54.5%) |
| Small (< 100) | 7 (14.9%) | 0 (0%) | 5 (10.9%) | 0 (0%) | 12 (12.9%) | 0 (0%) |
| **No. of form groups** |  |  |  |  |  |  |
| Median (IQR), number | 7 (3.75) | 6 (1) | 6 (2) | 8.5 (3.25) | 6 (3) | 7 (2.5) |
| Missing, no. (%) | 1 (2.1%) | 0 (0%) | 1 (2.2%) | 0 (0%) | 2 (2.2%) | 0 (0%) |
| **Ofsted rating** |  |  |  |  |  |  |
| Outstanding | 14 (29.8%) | 2 (40.0%) | 8 (17.4%) | 2 (33.3%) | 22 (23.7%) | 4 (36.4%) |
| Good | 24 (51.1%) | 1 (20.0%) | 29 (63.0%) | 2 (33.3%) | 53 (57.0%) | 3 (27.3%) |
| Requires Improvement | 6 (12.8%) | 1 (20.0%) | 4 (8.7%) | 1 (16.7%) | 10 (10.8%) | 2 (18.2%) |
| Serious Weaknesses | 0 (0%) | 0 (0%) | 1 (2.2%) | 0 (0%) | 1 (1.1%) | 0 (0%) |
| Special Measures | 3 (6.4%) | 1 (20.0%) | 3 (6.5%) | 1 (16.7%) | 6 (6.5%) | 2 (18.2%) |
| Missing, no. (%) | 0 (0%) | 0 (0%) | 1 (2.2%) | 0 (0%) | 1 (1.1%) | 0 (0%) |
| **Socioeconomic status** |  |  |  |  |  |  |
| eFSM, mean (SD), % | 17.7 (12.9) | 17.4 (13.9) | 17.0 (9.00) | 18.8 (5.04) | 17.3 (11.1) | 18.2 (9.53) |
| IMD, median (IQR), decile | 6 (6) | 8 (4) | 5 (5) | 4.5 (1.75) | 5 (5) | 5 (3.5) |
| **School location** |  |  |  |  |  |  |
| Rural town and fringe | 4 (8.5%) | 0 (0%) | 5 (10.9%) | 0 (0%) | 9 (9.7%) | 0 (0%) |
| Rural village | 0 (0%) | 1 (20.0%) | 0 (0%) | 0 (0%) | 0 (0%) | 1 (9.1%) |
| Urban city and town | 20 (42.6%) | 3 (60.0%) | 19 (41.3%) | 3 (50.0%) | 39 (41.9%) | 6 (54.5%) |
| Urban major conurbation | 23 (48.9%) | 1 (20.0%) | 22 (47.8%) | 3 (50.0%) | 45 (48.4%) | 4 (36.4%) |
|  |  |  |  |  |  |  |
| **PUPIL LEVEL** |  |  |  |  |  |  |
| No. participants | 8157 | 878 | 7860 | 1366 | 16017 | 2244 |
| Age, mean (SD), y | 12.5 (0.293) | 12.5 (0.294) | 12.5 (0.296) | 12.5 (0.297) | 12.5 (0.295) | 12.5 (0.296) |
| Female, no. (%) | 4495 (55.1%) | 340 (38.7%) | 4466 (56.8%) | 713 (52.2%) | 8961 (55.9%) | 1053 (46.9%) |
| eFSM, no. (%), yes | 1422 (17.4%) | 151 (17.2%) | 1243 (15.8%) | 252 (18.4%) | 2665 (16.6%) | 403 (18.0%) |

Abbreviations: eFSM = eligible for free school meals, IMD = index of multiple deprivation, IQR = interquartile range, SD = standard deviation, y = year

**Table 2. School level predictors of school drop-out pre-baseline assessment: analysis of deviance table (type II Wald Chi-square test)**

|  | **df** | **χ^2^** | ***P*** |
| --- | --- | --- | --- |
| Gender type | 2 | 0.73 | 0.69 |
| School type | 2 | 2.45 | 0.29 |
| Establishment type | 7 | 2.32 | 0.94 |
| School size | 2 | 0.97 | 0.62 |
| Number of form groups | 1 | 0.00 | 0.98 |
| Ofsted rating | 3 | 3.42 | 0.33 |
| Percent eFSM | 1 | 3.42 | 0.33 |
| IMD decile | 1 | 0.00 | 0.95 |
| School location | 3 | 3.72 | 0.29 |

Abbreviations: df = degrees of freedom, eFSM = eligible for free school meals, IMD = index of multiple deprivation

**Table 3. Pupil level predictors of school drop-out pre-baseline assessment (multilevel logistic regression with logit link function)**

|  | **OR** | **95% CI** | ***P*** |
| --- | --- | --- | --- |
| Sex (ref: female) | 1.18 | 0.001, 1043.13 | 0.96 |
| Age | 0.99 | 0.002, 452.8 | 1.00 |
| eFSM (ref: not eFSM) | 1.06 | 0.0002, 5870 | 0.99 |

Abbreviations: eFSM = eligible for free-school-meals, OR = odds ratio

**References**

1. Kosmidis I. Bias in parametric estimation: Reduction and useful side-effects. Wiley Interdiscip Rev Comput Stat. 2014;6(3):185–96.

2. Kosmidis I, Kenne Pagui EC, Sartori N. Mean and median bias reduction in generalized linear models. Stat Comput. 2020;30(1):43–59.
