# Supplementary material for: The effect of a one-year vigorous physical activity intervention on fitness, cognitive performance and mental health in young adolescents: the Fit to Study cluster randomised controlled trial
Source: Int J Behav Nutr Phys Act. 2021 Mar 31;18:47. doi: 10.1186/s12966-021-01113-y (PMC8011147; doi:10.1186/s12966-021-01113-y)
Supplement: Supplementary file 6 — Additional file 6:. Data cleaning [file 12966_2021_1113_MOESM6_ESM.docx]

**Additional file 6. Data cleaning**

An overview of participants who completed each assessment, per group and timepoint, is provided in Table 1. While cognitive assessments and online questionnaires were cleaned using a conservative cleaning approach, no cleaning has been applied to the 20MSR data.

**Table 1. Total number of participants who completed each assessment, pre and post cleaning, per group and timepoint.**

|  | **Intervention** | | | | | | | **Control** | | | | | | |
| --- | --- | --- | --- | --- | --- | --- | --- | --- | --- | --- | --- | --- | --- | --- |
|  | **Baseline** | | | **Posttest** | | | **Pre-post (cleaned)** | **Baseline** | | | **Posttest** | | | **Pre-post (cleaned)** |
|  | Raw | Clean | Excluded | Raw | Clean | Excluded |  | Raw | Clean | Excluded | Raw | Clean | Excluded |  |
| Fitness | 4927 |  |  | 3336 |  |  | 3049 | 5770 |  |  | 4778 |  |  | 4264 |
| Reaction time | 3610 | 3562 | 48 | 2504 | 2490 | 14 | 1500 | 4449 | 4378 | 71 | 3676 | 3649 | 27 | 2626 |
| Relational memory | 2564 | 2471 | 93 | 1936 | 1788 | 148 | 840 | 2954 | 2847 | 107 | 2936 | 2704 | 232 | 1445 |
| Two-back | 2395 | 2251 | 144 | 1938 | 1784 | 154 | 803 | 2928 | 2761 | 167 | 2937 | 2717 | 220 | 1468 |
| Flanker | 2730 | 2608 | 122 | 1930 | 1806 | 124 | 890 | 2983 | 2840 | 143 | 2916 | 2711 | 205 | 1489 |
| Task switching | 2374 | 2169 | 205 | 1831 | 1561 | 270 | 709 | 2817 | 2553 | 264 | 2819 | 2375 | 444 | 1221 |
| Questionnaire^a^ | 4753 | 4735 | 18 | 2578 | 2558 | 20 | 1899 | 5052 | 5040 | 12 | 3890 | 3856 | 34 | 2799 |

^a^Note: The values for the questionnaire may differ depending on the measure due sporadic missingness. Values presented here reflect the number of cases who completed the one or more sections of the questionnaire.

Given the large numbers of subjects that were removed from the dataset due to cleaning, we assessed whether participant characteristics could predict in or exclusion during cleaning using linear mixed effects models (with logit link function, available in the *lme4* package in R). Results of linear mixed effects models are presented in Table 2. Being male was predictive of exclusion due to cleaning for the majority of cognitive assessments (i.e. relational memory task, Flanker task, two-back task, colour-shape switch task, and questionnaire at post-intervention), as well as being eligible for FSM (relational memory task at baseline, the Flanker task at baseline, the two-back task and colour-shape switch task) and older age (questionnaire at baseline).

**Table 2.** **Models testing whether baseline characteristics predict removal during cleaning**

|  | **OR** | **95% CI** | ***p*** |
| --- | --- | --- | --- |
| **Relational memory task - baseline** |  |  |  |
| sex (reference = female) | 3.13 | 2.28, 4.3 | **< 0.01** |
| eFSM (reference = non eligible) | 2.42 | 1.71, 3.42 | **< 0.01** |
| age | 0.79 | 0.48, 1.29 | 0.35 |
| **Relational memory task - post-intervention** |  |  |  |
| sex (reference = female) | 3.19 | 2.47, 4.13 | **< 0.01** |
| eFSM (reference = non eligible) | 1.22 | 0.91, 1.64 | 0.19 |
| age | 1.01 | 0.69, 1.46 | 0.97 |
| **Reaction time task - baseline** |  |  |  |
| sex (reference = female) | 1.27 | 0.87, 1.86 | 0.22 |
| eFSM (reference = non eligible) | 1.13 | 0.69, 1.87 | 0.62 |
| age | 0.91 | 0.49, 1.68 | 0.76 |
| **Reaction time task - post-intervention** |  |  |  |
| sex (reference = female) | 1.34 | 0.71, 2.51 | 0.37 |
| eFSM (reference = non eligible) | 1.94 | 0.94, 4 | 0.07 |
| age | 1.31 | 0.45, 3.79 | 0.62 |
| **Flanker task - baseline** |  |  |  |
| sex (reference = female) | 3.14 | 2.37, 4.17 | **< 0.01** |
| eFSM (reference = non eligible) | 1.79 | 1.3, 2.46 | **< 0.01** |
| age | 0.91 | 0.59, 1.4 | 0.68 |
| **Flanker task - post-intervention** |  |  |  |
| sex (reference = female) | 2 | 1.54, 2.6 | **< 0.01** |
| eFSM (reference = non eligible) | 1.25 | 0.91, 1.71 | 0.16 |
| age | 0.86 | 0.58, 1.28 | 0.45 |
| **Two-back task - baseline** |  |  |  |
| sex (reference = female) | 1.64 | 1.28, 2.1 | **< 0.01** |
| eFSM (reference = non eligible) | 1.9 | 1.4, 2.56 | **< 0.01** |
| age | 1.16 | 0.78, 1.72 | 0.47 |
| **Two-back task - post-intervention** |  |  |  |
| sex (reference = female) | 1.85 | 1.45, 2.35 | **< 0.01** |
| eFSM (reference = non eligible) | 1.64 | 1.24, 2.16 | **< 0.01** |
| age | 0.76 | 0.52, 1.11 | 0.15 |
| **Colour-shape task switching - baseline** |  |  |  |
| sex (reference = female) | 2.24 | 1.81, 2.77 | **< 0.01** |
| eFSM (reference = non eligible) | 1.84 | 1.43, 2.38 | **< 0.01** |
| age | 1.01 | 0.72, 1.4 | 0.97 |
| **Colour-shape task switching - post-intervention** |  |  |  |
| sex (reference = female) | 2.48 | 2.05, 3 | **< 0.01** |
| eFSM (reference = non eligible) | 1.28 | 1.01, 1.62 | 0.04 |
| age | 0.9 | 0.68, 1.2 | 0.48 |
| **Questionnaire - baseline** |  |  |  |
| sex (reference = female) | 3.03 | 1.39, 6.59 | 0.01 |
| eFSM (reference = non eligible) | 1.54 | 0.62, 3.83 | 0.36 |
| age | 7.55 | 2.06, 27.61 | **< 0.01** |
| **Questionnaire - post-intervention** |  |  |  |
| sex (reference = female) | 6.15 | 2.8, 13.51 | **< 0.01** |
| eFSM (reference = non eligible) | 1.6 | 0.83, 3.09 | 0.16 |
| age | 0.66 | 0.25, 1.74 | 0.4 |

Abbreviations: CI = confidence intervals, eFSM = eligible for free school meals, OR = odds ratio
