# Supplementary material for: The effect of a one-year vigorous physical activity intervention on fitness, cognitive performance and mental health in young adolescents: the Fit to Study cluster randomised controlled trial
Source: Int J Behav Nutr Phys Act. 2021 Mar 31;18:47. doi: 10.1186/s12966-021-01113-y (PMC8011147; doi:10.1186/s12966-021-01113-y)
Supplement: Supplementary file 7 — Additional file 7:. Baseline school, PA and PE characteristics [file 12966_2021_1113_MOESM7_ESM.docx]

**Additional file 7. Baseline school, PA and PE characteristics**

School characteristics at baseline are presented in Table 1.

**Table 1. School characteristics at baseline**

|  | **Intervention** | **Control** |
| --- | --- | --- |
| **School level** |  |  |
| No. schools | 46 | 47 |
| Gender status, no. (%) |  |  |
| Co-ed | 37 (80.4%) | 38 (80.9%) |
| Female | 9 (19.6%) | 8 (17.0%) |
| Male | 0 (0%) | 1 (2.1%) |
| School type, no. (%) |  |  |
| Comprehensive to 16 | 7 (15.2%) | 9 (19.1%) |
| Comprehensive to 18 | 34 (73.9%) | 33 (70.2%) |
| Secondary Modern | 5 (10.9%) | 5 (10.6%) |
| Establishment type, no. (%) |  |  |
| Academy - Sponsor Led | 7 (15.2%) | 9 (19.1%) |
| Academy Converter | 2 (4.3%) | 1 (2.1%) |
| Academy Convertor - Mainstream | 16 (34.8%) | 15 (31.9%) |
| Community School | 8 (17.4%) | 7 (14.9%) |
| Foundation School | 5 (10.9%) | 5 (10.6%) |
| Free School - Mainstream | 3 (6.5%) | 2 (4.3%) |
| Voluntary aided school | 4 (8.7%) | 7 (14.9%) |
| Voluntary controlled school | 1 (2.2%) | 1 (2.1%) |
| School size, no. (%) |  |  |
| Large (> 200) | 14 (30.4%) | 13 (27.7%) |
| Medium (100-200) | 27 (58.7%) | 27 (57.4%) |
| Small (< 100) | 5 (10.9%) | 7 (14.9%) |
| Number of Year 7 form groups^a^, no. (%) |  |  |
| Median (range) | 6 (3- 12) | 7 (3-14) |
| Missing, no. (%) | 1 (2.2%) | 1 (2.1%) |
| Ofsted rating, no. (%) |  |  |
| Outstanding | 8 (17.4%) | 14 (29.8%) |
| Good | 29 (63.0%) | 24 (51.1%) |
| Requires Improvement | 4 (8.7%) | 6 (12.8%) |
| Serious Weaknesses | 1 (2.2%) | 0 (0%) |
| Special Measures | 3 (6.5%) | 3 (6.4%) |
| Missing, no. (%) | 1 (2.2%) | 0 (0%) |
| Socioeconomic status |  |  |
| Percent eFSM pupils, mean SD | 17.0 (9.00) | 17.7 (12.9) |
| IMD, median (range), decile | 5 (1-10) | 6 (1-10) |
| School location, no (%) |  |  |
| Rural town and fringe | 5 (10.9%) | 4 (8.5%) |
| Rural village | 0 (0%) | 0 (0%) |
| Urban city and town | 19 (41.3%) | 20 (42.6%) |
| Urban major conurbation | 22 (47.8%) | 23 (48.9%) |

Abbreviations: eFSM = eligible for free school meals, IMD = index of multiple deprivation, SD = standard deviation

**^a^** The number of Year 7 form groups per school were provided at the start of the trial. No information was collected on the number of Year 8 form groups; it was assumed that this number remained unchanged.

Physical activity characteristics of schools and pupils at baseline are presented in Table 2. Estimates of vigorous PA (VPA), moderate-to-vigorous PA (MVPA), moderate PA (MPA) and sedentary PA (SPA) were equivalent across the groups.

**Table 2. Physical activity characteristics of schools and pupils at baseline (available cases only)**

|  | **Intervention** | **Control** | **Overall** |
| --- | --- | --- | --- |
| **School level** |  |  |  |
| No. schools | 46 | 47 | 93 |
| Full PE lesson (1 hr) |  |  |  |
| VPA, mean (SD), min | 3.08 (0.939) | 3.31 (1.13) | 3.20 (1.04) |
| MVPA, mean (SD), min | 11.1 (2.16) | 11.2 (2.27) | 11.1 (2.20) |
| MPA, mean (SD), min | 7.99 (1.54) | 7.89 (1.52) | 7.94 (1.52) |
| SPA, mean (SD), min | 31.9 (4.26) | 31.9 (3.20) | 31.9 (3.75) |
| Active PE lesson (1 hr) |  |  |  |
| VPA, mean (SD), min | 3.93 (1.16) | 4.39 (1.60) | 4.16 (1.41) |
| MVPA, mean (SD), min | 13.7 (2.66) | 14.3 (3.00) | 14.0 (2.83) |
| MPA, mean (SD), min | 9.80 (1.93) | 9.90 (1.85) | 9.85 (1.88) |
| SPA, mean (SD), min | 26.9 (4.65) | 26.2 (3.96) | 26.6 (4.31) |
| Missing, no. (%) | 1 (2.2%) | 2 (4.3%) | 3 (3.2%) |
|  |  |  |  |
| **Pupil level** |  |  |  |
| No. pupils with PA data (total) | 4734 | 4965 | 9699 |
| PA (past week), no (%), days 60 min MVPA/day | |  |  |
| 0 | 156 (3.3%) | 129 (2.6%) | 285 (2.9%) |
| 1 | 239 (5.0%) | 239 (4.8%) | 478 (4.9%) |
| 2 | 468 (9.9%) | 412 (8.3%) | 880 (9.1%) |
| 3 | 728 (15.4%) | 730 (14.7%) | 1458 (15.0%) |
| 4 | 813 (17.2%) | 915 (18.4%) | 1728 (17.8%) |
| 5 | 839 (17.7%) | 877 (17.7%) | 1716 (17.7%) |
| 6 | 641 (13.5%) | 666 (13.4%) | 1307 (13.5%) |
| 7 | 850 (18.0%) | 997 (20.1%) | 1847 (19.0%) |
| Habitual PA (6 months), no. (%), 60 min MVPA/day | |  |  |
| Never | 80 (1.7%) | 78 (1.6%) | 158 (1.6%) |
| Rarely | 139 (2.9%) | 138 (2.8%) | 277 (2.9%) |
| Occasionally | 259 (5.5%) | 255 (5.1%) | 514 (5.3%) |
| Sometimes | 1152 (24.3%) | 1171 (23.6%) | 2323 (24.0%) |
| Frequently | 1059 (22.4%) | 1073 (21.6%) | 2132 (22.0%) |
| Usually | 1158 (24.5%) | 1265 (25.5%) | 2423 (25.0%) |
| Always | 887 (18.7%) | 985 (19.8%) | 1872 (19.3%) |

Abbreviations: No = number, MPA = moderate physical activity, MVPA = moderate-to-vigorous physical activity, PA = physical activity, PE= physical education, SD = standard deviation, SPA = sedentary physical activity, VPA = vigorous physical activity

**PE lessons across treatment and control schools**

Table 3 displays the average number of PE lessons, lesson duration and total minutes of PE per week in intervention and control schools.

**Table 3. PE lesson characteristics across intervention and control schools**

|  | **Intervention (n=46)** | **Control (n=47)** | **Overall (93)** |
| --- | --- | --- | --- |
| Number of PE lessons per week |  |  |  |
| Mean (SD) | 1.61 (0.493) | 1.59 (0.498) | 1.60 (0.493) |
| Median (range) | 2 (1 - 2) | 2 (1 - 2) | 2 (1 - 2) |
| Missing, no. (%) | 2 (4.3%) | 1 (2.1%) | 3 (3.2%) |
| Lesson duration, min |  |  |  |
| Mean (SD) | 72.3 (24.4) | 72.2 (20.1) | 72.3 (22.2) |
| Median (range) | 60 (50, 150) | 60 (47.5, 120) | 60 (47.5, 150) |
| Missing, no. (%) | 1 (2.2%) | 1 (2.1%) | 2 (2.2%) |
| Total min of PE per week |  |  |  |
| Mean (SD) | 110 (31.0) | 107 (20.7) | 109 (26.2) |
| Median (range) | 120 (50, 240) | 120 (47.5, 150) | 120 (7.5, 240) |
| Missing, no. (%) | 2 (4.3%) | 1 (2.1%) | 3 (3.2%) |

Abbreviations: SD = standard deviation, PE = physical education
