# Supplementary material for: The effect of a one-year vigorous physical activity intervention on fitness, cognitive performance and mental health in young adolescents: the Fit to Study cluster randomised controlled trial
Source: Int J Behav Nutr Phys Act. 2021 Mar 31;18:47. doi: 10.1186/s12966-021-01113-y (PMC8011147; doi:10.1186/s12966-021-01113-y)
Supplement: Supplementary file 8 — Additional file 8:. Additional information for primary outcome analyses [file 12966_2021_1113_MOESM8_ESM.docx]

**Additional file 8. Primary outcomes**

***Fully adjusted models: multiply imputed data***

Table 1 displays the (un)standardized mean differences between the groups at post intervention (adjusted for baseline values of the outcome measure, school gender type and *when* and *where* assessments were completed at baseline and posttest).

**Table 1. The effect of the intervention on secondary outcomes (fully adjusted models)**

|  | **Adjusted mean difference^a^ (95% CI)** | |
| --- | --- | --- |
|  | Unstandardized | Standardized^b^ |
| 20MSR |  |  |
| Fitness, laps | 0.43 (-2.61, 3.47) | 0.02 (-0.12, 0.16) |
| Reaction time task |  |  |
| RT^c^, ms | 3.85 (-6.8, 14.5) | 0.04 (-0.07, 0.15) |
| Relational memory task |  |  |
| Accuracy, % | -1.37 (-3, 0.27) | -0.1 (-0.23, 0.02) |
| Two-back task |  |  |
| Accuracy, % | -1.04 (-3.47, 1.38) | -0.05 (-0.17, 0.07) |
| RT^c^, ms | -5.84 (-24.79, 13.1) | -0.03 (-0.14, 0.07) |
| Flanker task |  |  |
| Accuracy congruent, % | -0.8 (-2.28, 0.68) | -0.05 (-0.14, 0.04) |
| Accuracy incongruent, % | -1.14 (-2.97, 0.69) | -0.06 (-0.15, 0.03) |
| RT^c^ congruent, ms | -2.75 (-10.63, 5.13) | -0.03 (-0.13, 0.06) |
| RT^c^ incongruent, ms | -6.04 (-15.76, 3.68) | -0.06 (-0.14, 0.03) |
| Colour-shape switching task |  |  |
| Accuracy non-switch, % | -0.83 (-2.7, 1.05) | -0.05 (-0.15, 0.06) |
| Accuracy switch, % | -0.42 (-2.26, 1.43) | -0.02 (-0.13, 0.08) |
| RT^c^ non-switch, ms | -5.75 (-34.54, 23.05) | -0.02 (-0.11, 0.07) |
| RT^c^ switch, ms | -14.63 (-68.45, 39.19) | -0.03 (-0.12, 0.07) |
| Psychosocial problems |  |  |
| Internalising score^c^ | 0.19 (-0.19, 0.56) | 0.05 (-0.05, 0.15) |
| Externalising score^c^ | 0.24 (-0.13, 0.6) | 0.06 (-0.03, 0.16) |
| Self-esteem |  |  |
| Global | -0.05 (-0.22, 0.12) | -0.05 (-0.21, 0.11) |
| Physical | -0.02 (-0.22, 0.19) | -0.01 (-0.16, 0.13) |

Abbreviations: CRF = cardiorespiratory fitness, ms = millisecond, RT = reaction time

^a^ Adjusted mean difference, adjusted for baseline values of the outcome variable, school gender type, location and date variables, and including a random intercept term across schools

^b^ The outcome was standardized (mean = 0, SD = 1), prior to fitting the fully adjusted model

^c^ Lower scores represent better performance

***Sensitivity analysis: complete-cases***

Table 2 displays the mean scores on all outcome measures by treatment group, as well as the (un)standardized mean differences between the groups at post intervention (adjusted for baseline values of the outcome measure and school gender type).

**Table 2. Mean scores for outcome measures by group, with (un)standardized mean differences**

|  | **Intervention group** | | | **Control group** | | | **Adjusted mean difference^a^ (95% CI)** | |
| --- | --- | --- | --- | --- | --- | --- | --- | --- |
|  | N | Baseline | Post | N | Baseline | Post | Unstandardized | Standardized^b^ |
|  |  | M(SD) | M(SD) |  | M(SD) | M(SD) |  |  |
| 20MSR | | | | | | | | |
| CRF, laps | 3049 | 38.6 (21.3) | 43.9 (21.9) | 4264 | 38.3 (19.9) | 42.5 (22.1) | 1.12 (-2.03, 4.26) | 0.05 (-0.09, 0.19) |
| Reaction time task | | | | | | | | |
| RT, ms^c^ | 1500 | 375 (89.2) | 378 (95.1) | 2626 | 372 (80.3) | 371 (91.6) | 6.63 (-5.26, 18.52) | 0.034 (-0.05, 0.12) |
| Relational memory task | | | | | | | | |
| Accuracy, % | 840 | 60.9 (12.0) | 62.6 (12.2) | 1445 | 61.8 (12.0) | 64.0 (12.9) | -1.33 (-3.22, 0.56) | -0.11 (-0.25, 0.04) |
| Two-back task | | | | | | | | |
| Accuracy, % | 803 | 57.2 (17.2) | 61.1 (19.5) | 1468 | 57.9 (17.9) | 62.3 (19.8) | -1.1 (-4.1, 1.9) | -0.05 (-0.20, 0.09) |
| RT, ms^c^ | 803 | 881 (160) | 824 (162) | 1468 | 871 (160) | 816 (168) | 1.77 (-20.1, 23.66) | 0.01 (-0.12, 0.14) |
| Flanker task |  |  |  |  |  |  |  |  |
| Accuracy congruent, % | 890 | 85.1 (16.3) | 88.2 (14.5) | 1489 | 85.4 (15.2) | 88.6 (14.6) | -0.48 (-2.09, 1.14) | -0.03 (-0.13, 0.07) |
| Accuracy incongruent, % | 890 | 63.9 (20.2) | 67.9 (19.0) | 1489 | 63.7 (19.6) | 68.5 (18.7) | -0.94 (-2.87, 0.99) | -0.05 (-0.15, 0.05) |
| RT congruent, ms^c^ | 890 | 492 (90.3) | 477 (76.6) | 1489 | 494 (94.1) | 478 (78.1) | -1.29 (-8.24, 5.67) | -0.02 (-0.10, 0.07) |
| RT incongruent, ms^c^ | 890 | 559 (124) | 543 (95.0) | 1489 | 561 (123) | 547 (102) | -4.73 (-12.69, 3.22) | -0.04 (-0.12, 0.03) |
| Colour-shape switching task | | | | | | | | |
| Accuracy non-switch, % | 709 | 77.8 (16.9) | 80.9 (16.5) | 1221 | 78.1 (16.9) | 82.3 (16.2) | -1.34 (-3.52, 0.84) | -0.08 (-0.21, 0.05) |
| Accuracy switch, % | 709 | 73.7 (16.5) | 77.5 (16.3) | 1221 | 74.4 (16.8) | 78.2 (16.7) | -0.56 (-2.64, 1.51) | -0.03 (-0.15, 0.09) |
| RT non-switch, ms^c^ | 709 | 1090 (345) | 967 (296) | 1221 | 1080 (327) | 953 (294) | 9.27 (-25.22, 43.75) | 0.03 (-0.08, 0.14) |
| RT switch, ms^c^ | 709 | 1540 (586) | 1400 (497) | 1221 | 1510 (549) | 1390 (501) | -13.9 (-72.47, 44.67) | -0.03 (-0.14, 0.08) |
| Psychosocial problems | | | | | | | | |
| Internalising score^c^ | 1899 | 4.97 (3.47) | 5.31 (3.59) | 2795 | 5.16 (3.48) | 5.38 (3.57) | 0.02 (-0.27, 0.31) | 0.004 (-0.08, 0.09) |
| Externalising score^c^ | 1899 | 5.75 (3.59) | 6.16 (3.58) | 2790 | 5.88 (3.64) | 6.19 (3.75) | 0.03 (-0.27, 0.33) | 0.009 (-0.07, 0.09) |
| Self-esteem | | | | | | | | |
| Global | 1886 | 4.45 (0.896) | 4.32 (0.967) | 2683 | 4.50 (0.865) | 4.35 (0.951) | 0.01 (-0.07, 0.08) | 0.01 (-0.07, 0.09) |
| Physical | 1886 | 4.39 (1.27) | 4.07 (1.33) | 2684 | 4.44 (1.25) | 4.13 (1.32) | -0.02 (-0.09, 0.05) | -0.01 (-0.06, 0.04) |

Abbreviations: CRF = cardiorespiratory fitness, ms = millisecond, RT = reaction time

^a^ Adjusted mean difference, adjusted for baseline values of the outcome variable and school gender type, and including a random intercept term across schools

^b^ The outcome was standardized (mean = 0, SD = 1), prior to fitting the baseline and stratification-variable adjusted model

^c^ Lower scores represent better performance

***Distributions***


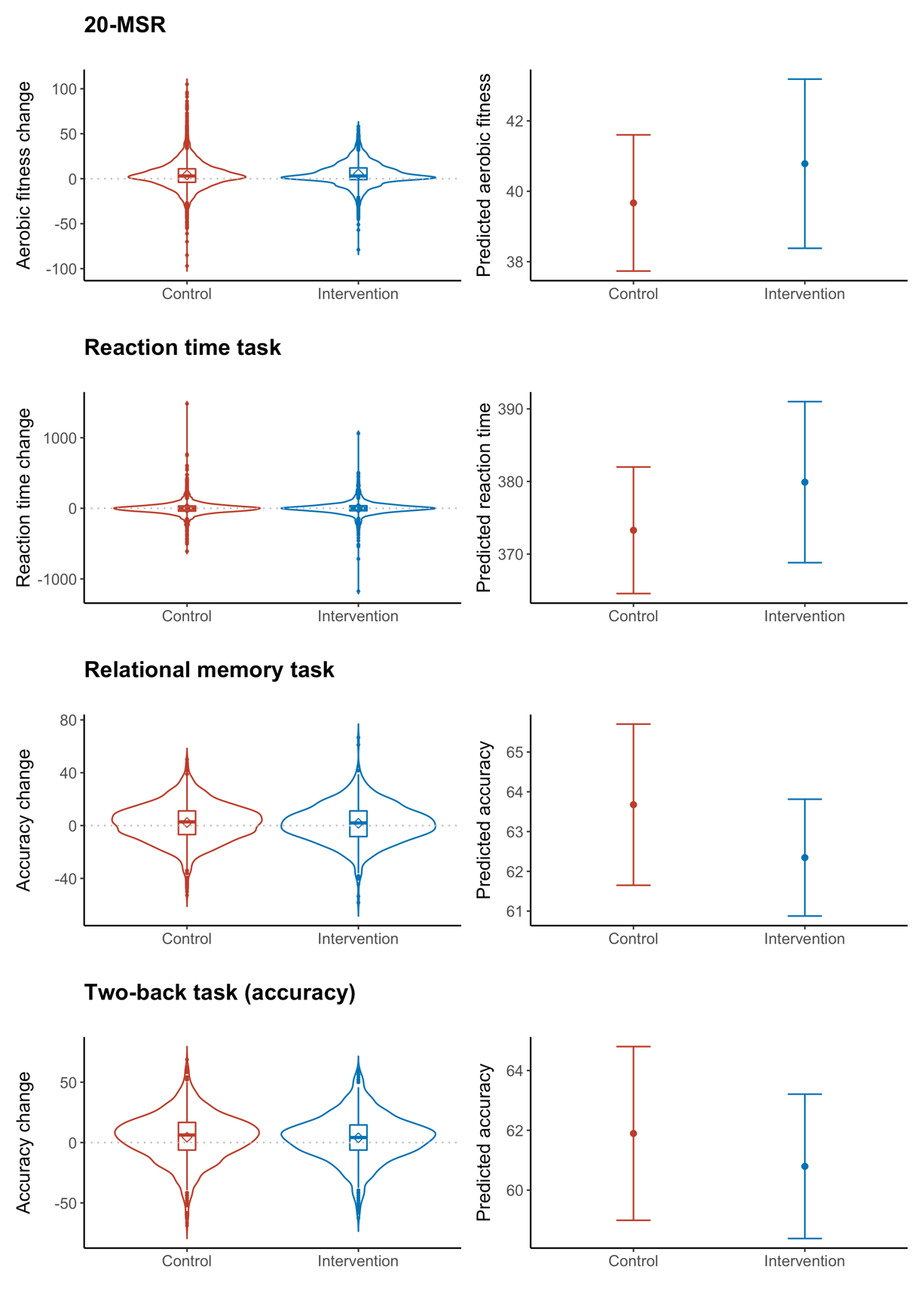
The distributions in change of outcome measures (post – pre test scores) are visualised (left column), alongside the predicted outcomes (i.e. marginal means, at post-test; right column), using the primary intention-to-treat model. The predicted (marginal) effects are displayed with 95% confidence intervals, based on the standard error, and created with the *ggeffects* package in R (1). All plots are based on available (complete-cases) only.

**
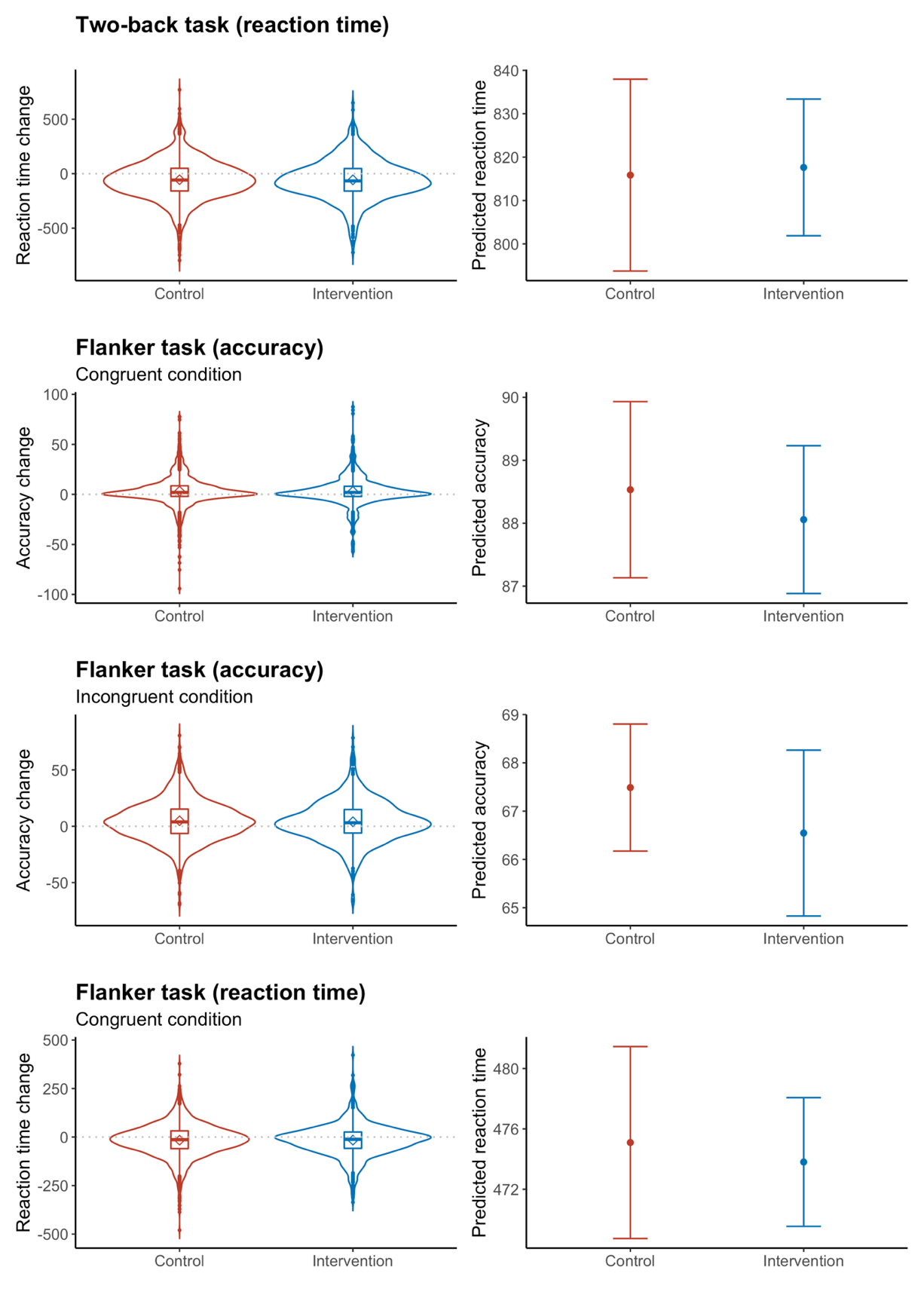
**


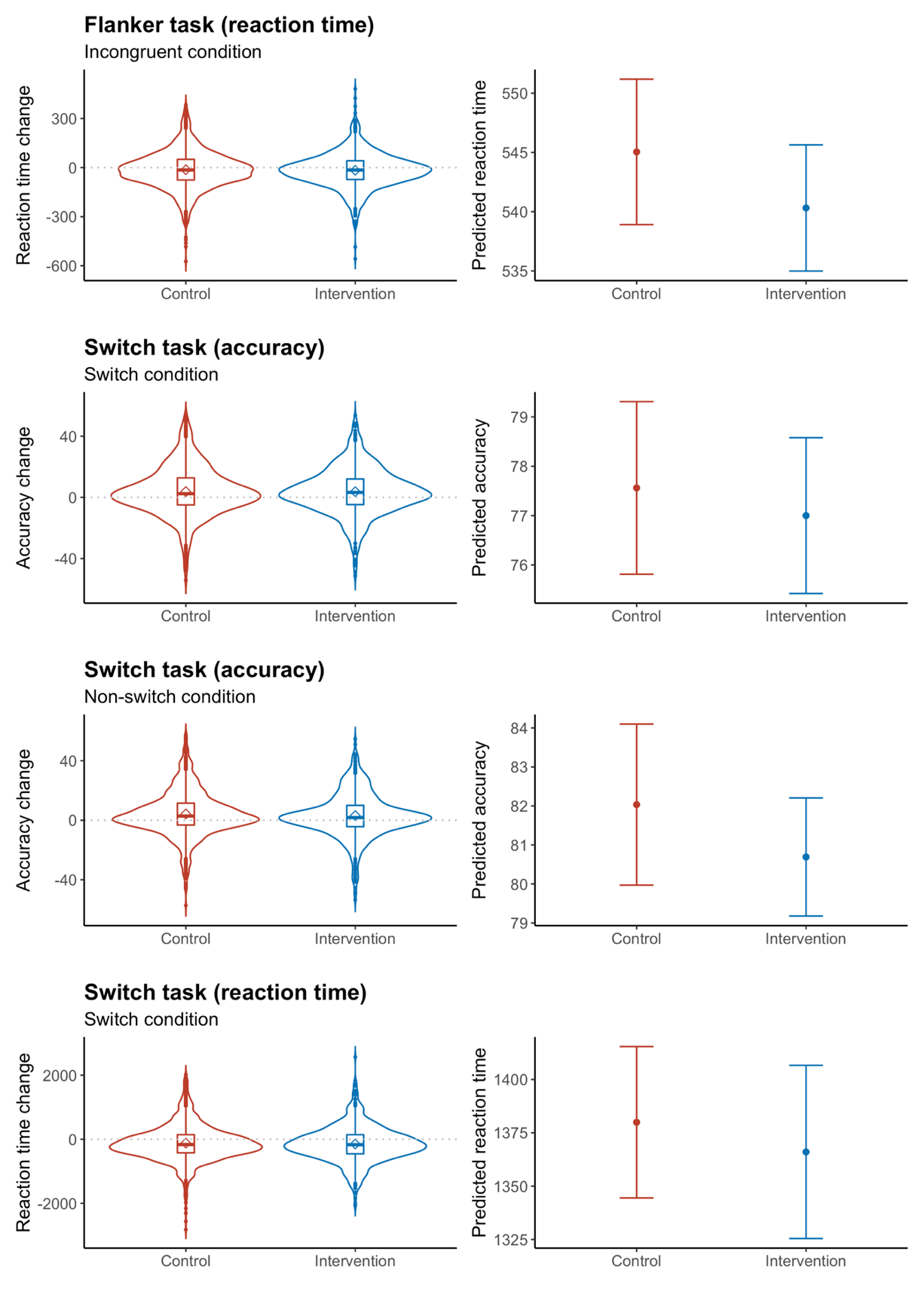


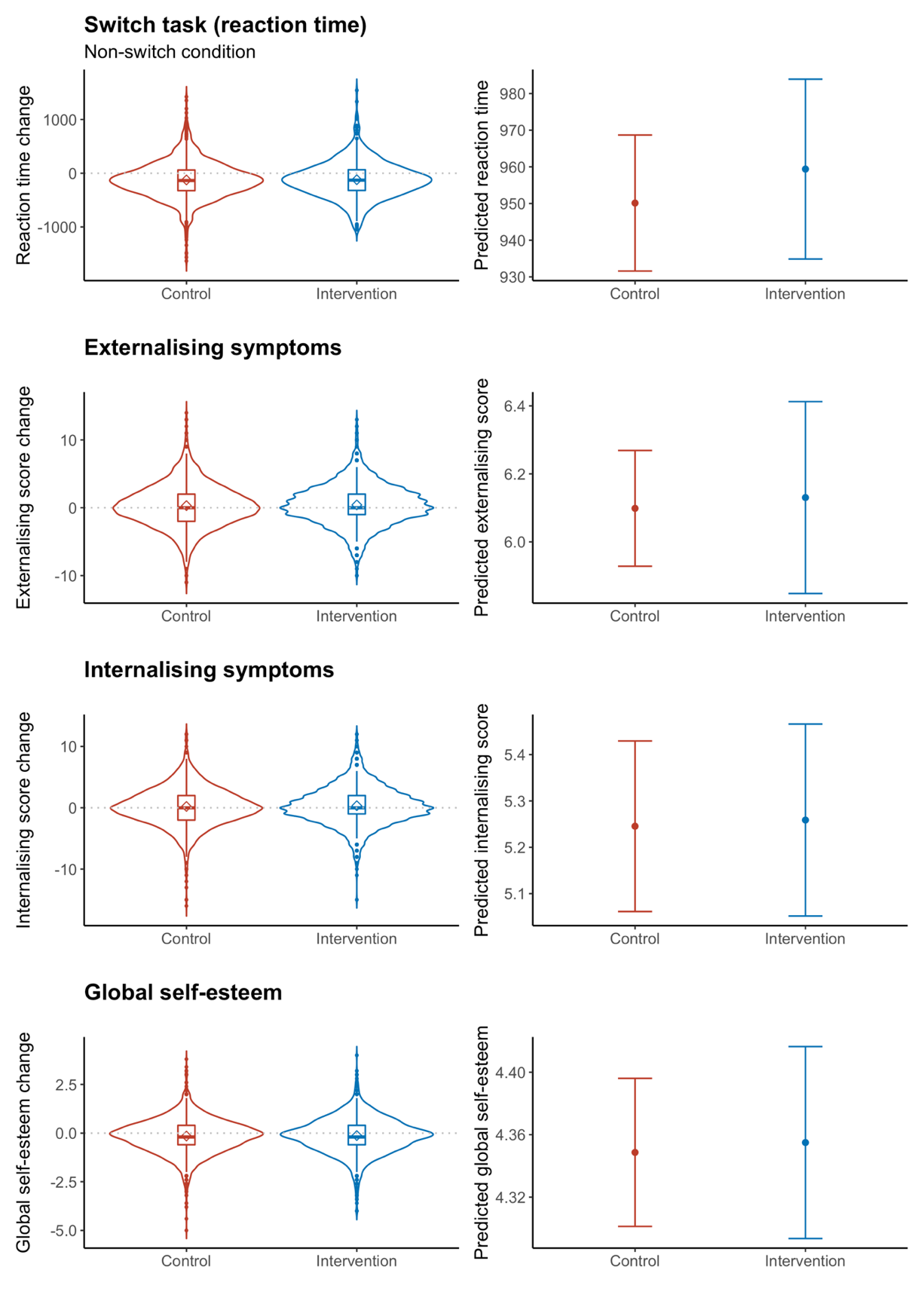


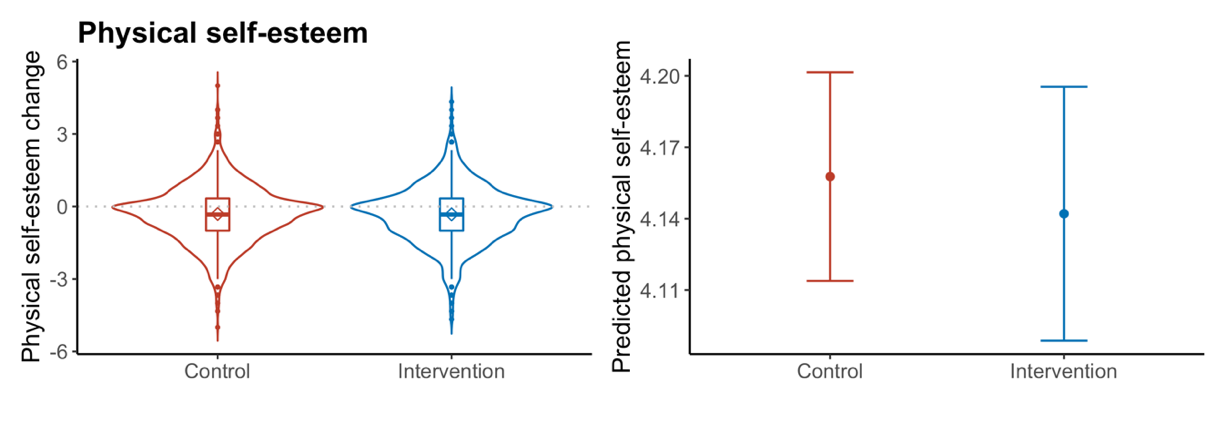


***Fully adjusted models***

Table 3 presents the intervention standardized and unstandardized differences for the fully adjusted models in which we additionally controlled for location (school or home) and date on which the tasks were completed (summer, holidays and autumn; for fitness: summer and autumn only).

**Table 3. The effect of the intervention on secondary outcomes (fully adjusted models)**

|  | **N** | | **Adjusted mean difference^a^ (95% CI)** | |
| --- | --- | --- | --- | --- |
|  | N_total_ | N_clusters_ | Unstandardized | Standardized^b^ |
| 20MSR |  |  |  |  |
| Fitness, laps | 7313 | 55 | 1.02 (-2.27, 4.3) | 0.05 (-0.1, 0.2) |
| Reaction time task |  |  |  |  |
| RT^c^, ms | 4126 | 55 | 8.17 (-2.63, 18.96) | 0.09 (-0.03, 0.2) |
| Relational memory task |  |  |  |  |
| Accuracy, % | 2285 | 55 | -1.41 (-3.35, 0.54) | -0.11 (-0.27, 0.04) |
| Two-back task |  |  |  |  |
| Accuracy, % | 2271 | 55 | -1.71 (-4.86, 1.44) | -0.08 (-0.24, 0.07) |
| RT^c^, ms | 2271 | 55 | -3.44 (-26.45, 19.56) | -0.02 (-0.16, 0.12) |
| Flanker task |  |  |  |  |
| Accuracy congruent, % | 2379 | 55 | -0.5 (-2.26, 1.26) | -0.03 (-0.14, 0.08) |
| Accuracy incongruent, % | 2379 | 55 | -1.16 (-3.13, 0.8) | -0.06 (-0.16, 0.04) |
| RT^c^ congruent, ms | 2379 | 55 | -2.55 (-10.08, 4.98) | -0.03 (-0.13, 0.06) |
| RT^c^ incongruent, ms | 2379 | 55 | -6.26 (-15.09, 2.56) | -0.06 (-0.14, 0.02) |
| Colour-shape switching task |  |  |  |  |
| Accuracy non-switch, % | 1930 | 55 | -1.82 (-3.88, 0.23) | -0.11 (-0.23, 0.01) |
| Accuracy switch, % | 1930 | 55 | -1.1 (-2.95, 0.75) | -0.06 (-0.17, 0.04) |
| RT^c^ non-switch, ms | 1930 | 55 | 13.96 (-18.26, 46.17) | 0.05 (-0.06, 0.15) |
| RT^c^ switch, ms | 1930 | 55 | -21.36 (-77.12, 34.4) | -0.04 (-0.14, 0.06) |
| Psychosocial problems |  |  |  |  |
| Internalising score^c^ | 4660 | 52 | 0.04 (-0.27, 0.35) | 0.01 (-0.08, 0.1) |
| Externalising score^c^ | 4655 | 52 | 0.08 (-0.2, 0.36) | 0.02 (-0.05, 0.1) |
| Self-esteem |  |  |  |  |
| Global | 4536 | 52 | 0 (-0.08, 0.08) | 0 (-0.08, 0.08) |
| Physical | 4537 | 52 | -0.02 (-0.09, 0.06) | -0.01 (-0.07, 0.04) |

Abbreviations: CRF = cardiorespiratory fitness, ms = millisecond, RT = reaction time

^a^ Adjusted mean difference, adjusted for baseline values of the outcome variable and school gender type, and including a random intercept term across schools

^b^ The outcome was standardized (mean = 0, SD = 1), prior to fitting the baseline and stratification-variable adjusted model

^c^ Lower scores represent better performance

**References**

1. Lüdecke D. ggeffects: Tidy Data Frames of Marginal Effects from Regression Models. J Open Source Softw. 2018;3(26):772.
