# Supplementary material for: The effect of a one-year vigorous physical activity intervention on fitness, cognitive performance and mental health in young adolescents: the Fit to Study cluster randomised controlled trial
Source: Int J Behav Nutr Phys Act. 2021 Mar 31;18:47. doi: 10.1186/s12966-021-01113-y (PMC8011147; doi:10.1186/s12966-021-01113-y)
Supplement: Supplementary file 9 — Additional file 9:. Additional information for subgroup analyses [file 12966_2021_1113_MOESM9_ESM.docx]

**Additional File 9. Subgroup analysis**

We examined whether the effect of the intervention on fitness, cognitive outcomes and mental health was moderated by sex, socioeconomic status (SES, indicated by eFSM status) or baseline fitness levels (dichotomous: high or low). Table 1 presents the standardized and unstandardized estimates of the interaction terms of each subgroup analysis, per outcome measure, on multiply imputed data. No significant moderation of sex, socioeconomic status or baseline fitness levels was observed following correction for multiple comparisons (alpha = 0.002, with 24 tests). In uncorrected analyses, baseline fitness levels significantly moderated the effect of the intervention on fitness (uncorrected *p* = 0.03), with better performance (i.e. higher number of laps) for intervention participants with high baseline fitness levels. However, no significant effect of the intervention was observed when the model was run in each subgroup (*p* > 0.05). Moreover, eFSM significantly moderated the effect of the intervention on processing speed (uncorrected *p* = 0.04), with worse performance (i.e. higher reaction times) for intervention participants that were eligible for free school meals (eFSM). A small significant effect of the intervention was found when the model was run in the eFSM subgroup only, with worse performance (i.e. higher reaction times) for pupils in the intervention group (*p* = 0.04).

A multigroup model, based on Figure 2 (main manuscript), was used to assess whether the effect of the intervention on the EF latent variable was equivalent for those classified as fit or unfit, high or low SES and males or females. We first checked for metric invariance as a function of each moderator (baseline fitness, SES and sex). We obtained full metric invariance between the groups for the fitness grouping (equal unstandardized factor loadings; test of the difference between configural and metric invariance χ^2^ (8) = 10.32, *p* = 0.24) and socioeconomic status (equal unstandardized factor loadings; test of the difference between configural and metric invariance χ^2^ (8) = 5.88, *p* = 0.66), but not for sex. The subgroup analysis by sex was therefore not pursued any further.

We next ran a model in which the regression coefficients from intervention to EF at posttest were freely estimated for high and low fitness groups, in this model the unstandardized regression coefficients for the intervention did not differ significantly between groups (Wald test χ^2^ (1) = 0.28, *p* = 0.60). The intervention produced no statistically significant change in EF in either group (unfit: SMD = -0.02; 95% CI [-0.19, 0.14]; fit: SMD = 0.01; 95% CI [-0.16, 0.18]) and the size of these effects did not differ significantly between fitness groups.

The multigroup ANCOVA model in which the regression coefficients from intervention to EF at posttest were freely estimated for the high and low SES (i.e. non eFSM and eFSM respectively) groups showed that the unstandardized regression coefficients differed significantly between groups (Wald test χ^2^ (1) = 5.60, *p* = 0.02). However, the intervention produced no statistically significant change in EF in either group (non eFSM: SMD = 0.03; 95% CI [-0.13, 0.19]; eFSM: SMD = -0.21; 95% CI [-0.43, 0.01]) although the intervention in the FSM group tended to impair EF skills at posttest.

**Table 1. Subgroup analysis, examining whether sex, eFSM or baseline fitness moderate the effect of the intervention on fitness (high / low), cognitive and mental health outcomes**

| outcome | moderator | Unstandardized | | Standardized | | ***P*_unc_^b^** |
| --- | --- | --- | --- | --- | --- | --- |
|  |  | Estimate^a^ | 95% CI | Estimate^a^ | 95% CI |  |
| 20MSR | sex | -2.13 | -5.12, 0.86 | -0.1 | -0.23, 0.04 | 0.16 |
| 20MSR | eFSM | -1.73 | -4.22, 0.76 | -0.08 | -0.19, 0.03 | 0.17 |
| 20MSR | fitness (t_0_) | 2.97 | 0.34, 5.6 | 0.13 | 0.02, 0.25 | **0.03** |
| Relational memory task, accuracy | sex | -0.54 | -2.38, 1.29 | -0.04 | -0.18, 0.1 | 0.56 |
| Relational memory task, accuracy | eFSM | 0.43 | -2.1, 2.95 | 0.03 | -0.16, 0.23 | 0.74 |
| Relational memory task, accuracy | fitness (t_0_) | -0.88 | -2.61, 0.85 | -0.07 | -0.2, 0.06 | 0.31 |
| Reaction time task, rt | sex | -2.82 | -14.8, 9.17 | -0.03 | -0.15, 0.09 | 0.64 |
| Reaction time task, rt | eFSM | 18.09 | 1.18, 35 | 0.19 | 0.01, 0.36 | **0.04** |
| Reaction time task, rt | fitness (t_0_) | 5.59 | -5.73, 16.91 | 0.06 | -0.06, 0.17 | 0.33 |
| Internalising score | sex | -0.09 | -0.53, 0.34 | -0.03 | -0.14, 0.09 | 0.68 |
| Internalising score | eFSM | 0.06 | -0.48, 0.61 | 0.02 | -0.13, 0.17 | 0.82 |
| Internalising score | fitness (t_0_) | 0.02 | -0.34, 0.38 | 0.01 | -0.09, 0.11 | 0.91 |
| Externalising score | sex | -0.02 | -0.43, 0.4 | 0 | -0.11, 0.11 | 0.94 |
| Externalising score | eFSM | 0.17 | -0.34, 0.68 | 0.04 | -0.09, 0.18 | 0.52 |
| Externalising score | fitness (t_0_) | -0.06 | -0.42, 0.3 | -0.02 | -0.11, 0.08 | 0.75 |
| Global self-esteem | sex | 0.11 | -0.02, 0.24 | 0.11 | -0.02, 0.24 | 0.11 |
| Global self-esteem | eFSM | -0.06 | -0.24, 0.13 | -0.06 | -0.24, 0.13 | 0.56 |
| Global self-esteem | fitness (t_0_) | -0.05 | -0.19, 0.1 | -0.05 | -0.19, 0.09 | 0.52 |
| Physical self-esteem | sex | 0.12 | -0.05, 0.29 | 0.09 | -0.03, 0.21 | 0.16 |
| Physical self-esteem | eFSM | -0.05 | -0.27, 0.18 | -0.03 | -0.2, 0.13 | 0.69 |
| Physical self-esteem | fitness (t_0_) | -0.07 | -0.25, 0.1 | -0.05 | -0.18, 0.07 | 0.41 |

Abbreviations: 20MSR = 20 meter shuttle run, CI = confidence interval, eFSM = eligible for free school meals, rt = reaction time

^a^ Displays the estimate of the interaction term between randomised group (reference = control) and baseline fitness status (reference = low fit), sex (reference = female), or eFSM (reference = not eFSM)

^b^ The alpha level was adjusted for multiple comparisons (24 tests) to be 0.002; uncorrected p-values are displayed

***Sensitivity analysis: complete-cases***

Table 2 presents the standardized and unstandardized estimates of the interaction terms of each subgroup analysis, per outcome measure. No significant moderation of sex, socioeconomic status or baseline fitness levels was observed following correction for multiple comparisons, in line with the primary multiply imputed analyses (Table 1). Baseline fitness levels significantly moderated the effect of the intervention on processing speed (uncorrected *p* = 0.02), with better performance (i.e. low reaction time) for control participants with high baseline fitness levels. A small significant effect of the intervention was observed when the model was run in the group with high baseline fitness levels, showing worse performance (i.e. higher reaction times) in the intervention compared to control group (*p* = 0.047).

**Table 2. Subgroup analysis, examining whether sex, eFSM or baseline fitness moderate the effect of the intervention on fitness (high / low), cognitive and mental health outcomes**

| **outcome** | **moderator** | **N** | **Unstandardized** | | **Standardized** | | ***P*_unc_^b^** |
| --- | --- | --- | --- | --- | --- | --- | --- |
|  |  |  | Estimate^a^ | 95% CI | Estimate^a^ | 95% CI |  |
| 20MSR | sex | 7313 | -2.51 | -7.17, 2.16 | -0.11 | -0.33, 0.1 | 0.28 |
| 20MSR | eFSM | 7313 | -2.07 | -4.6, 0.46 | -0.09 | -0.21, 0.02 | 0.1 |
| 20MSR | fitness (t_0_) | 7313 | 1.27 | -2.87, 5.41 | 0.06 | -0.13, 0.25 | 0.54 |
| Relational memory task, accuracy | sex | 2285 | -1.47 | -4.37, 1.42 | -0.12 | -0.35, 0.11 | 0.3 |
| Relational memory task, accuracy | eFSM | 2285 | 2.77 | -1.81, 7.35 | 0.22 | -0.14, 0.58 | 0.22 |
| Relational memory task, accuracy | fitness (t_0_) | 1981 | -2.05 | -4.9, 0.79 | -0.16 | -0.39, 0.06 | 0.15 |
| Reaction time task, rt | sex | 4126 | 4.26 | -18.27, 26.8 | 0.04 | -0.19, 0.28 | 0.7 |
| Reaction time task, rt | eFSM | 4126 | -2.16 | -27.54, 23.21 | -0.02 | -0.29, 0.24 | 0.86 |
| Reaction time task, rt | fitness (t_0_) | 3594 | 15.44 | 2.62, 28.26 | 0.16 | 0.03, 0.3 | **0.02** |
| Internalising score | sex | 4694 | 0.01 | -0.46, 0.49 | 0 | -0.13, 0.14 | 0.96 |
| Internalising score | eFSM | 4694 | 0.16 | -0.38, 0.71 | 0.05 | -0.11, 0.2 | 0.54 |
| Internalising score | fitness (t_0_) | 4024 | 0.14 | -0.23, 0.51 | 0.04 | -0.07, 0.14 | 0.46 |
| Externalising score | sex | 4689 | 0.15 | -0.3, 0.61 | 0.04 | -0.08, 0.17 | 0.49 |
| Externalising score | eFSM | 4689 | 0.18 | -0.32, 0.68 | 0.05 | -0.09, 0.18 | 0.46 |
| Externalising score | fitness (t_0_) | 4019 | -0.15 | -0.56, 0.26 | -0.04 | -0.15, 0.07 | 0.46 |
| Global self-esteem | sex | 4569 | 0.06 | -0.06, 0.19 | 0.07 | -0.06, 0.2 | 0.3 |
| Global self-esteem | eFSM | 4569 | -0.09 | -0.31, 0.12 | -0.1 | -0.32, 0.12 | 0.37 |
| Global self-esteem | fitness (t_0_) | 3916 | -0.05 | -0.17, 0.07 | -0.05 | -0.18, 0.08 | 0.42 |
| Physical self-esteem | sex | 4570 | 0.09 | -0.05, 0.24 | 0.07 | -0.04, 0.18 | 0.21 |
| Physical self-esteem | eFSM | 4570 | -0.13 | -0.36, 0.09 | -0.1 | -0.27, 0.07 | 0.23 |
| Physical self-esteem | fitness (t_0_) | 3917 | -0.09 | -0.26, 0.09 | -0.06 | -0.2, 0.07 | 0.33 |

Abbreviations: 20MSR = 20 meter shuttle run, CI = confidence interval, eFSM = eligible for free school meals, rt = reaction time

^a^ Displays the estimate of the interaction term between randomised group (reference = control) and baseline fitness status (reference = low fit), sex (reference = female), or eFSM (reference = not eFSM)

^b^ The alpha level was adjusted for multiple comparisons (25 tests) to be 0.002; uncorrected p-values are displayed
