# Supplementary material for: The effect of a one-year vigorous physical activity intervention on fitness, cognitive performance and mental health in young adolescents: the Fit to Study cluster randomised controlled trial
Source: Int J Behav Nutr Phys Act. 2021 Mar 31;18:47. doi: 10.1186/s12966-021-01113-y (PMC8011147; doi:10.1186/s12966-021-01113-y)
Supplement: Supplementary file 10 — Additional file 10:. Additional information for change-change analyses [file 12966_2021_1113_MOESM10_ESM.docx]

**ADDITIONAL FILE 10. Change-change analysis**

We explored whether a change in CRF was related to a change in cognitive or mental health outcomes, and whether this relationship was moderated by intervention status, adjusted for age, sex, eFSM, school gender type (boys-only, girls-only or co-eductional), and location (home or school) and period of completion (summer, holidays or autumn for cognitive and mental health measures; summer and autumn for CRF) of assessments. Tables 1 and 2 show the model terms and estimates for respectively, cognitive and mental health outcomes on multiply imputed cases. The alpha level was adjusted for multiple comparisons (alpha = 0.01 with 5 comparisons). Model 1 contained an interaction term between CRF-change and the intervention indicator. If the interaction was not significant, the interaction term was removed and the model was reassessed (model 2). Intervention status did not significantly moderate relationships between changes in CRF and changes in cognitive or mental health outcomes, and changes in CRF were not significantly related with any of the outcomes. In uncorrected analyses, the relationship between changes in CRF and changes in processing speed was moderated by intervention (*p* = 0.04). However, no significant relationships were observed when the model was run in each subgroup (*p* > 0.05).

**Table 1. Models examining whether a change in CRF is related to a change in cognitive outcomes**

|  | **Relational memory** | | | **Processing speed** | | |
| --- | --- | --- | --- | --- | --- | --- |
|  | ß | 95% CI | *p* | ß | 95% CI | *p* |
| Model 1: CRF change by group (ref = control) | 0.06 | -0.02, 0.14 | 0.13 | -0.57 | -1.12, -0.03 | **0.04** |
| Model 2: CRF change^a^ | -1.39 | -3.02, 0.24 | 0.10 | 5.07 | -6.28, 16.43 | 0.38 |

Abbreviations: CI = confidence interval, CRF = cardiorespiratory fitness

^a^ Model 2 did not contain an interaction term between CRF change and intervention group

**Table 2. Models examining whether a change in CRF is related to a change in mental health outcomes**

|  | **Internalising symptoms** | | | **Externalising symptoms** | | | **Global self-esteem** | | | **Physical self-esteem** | | |
| --- | --- | --- | --- | --- | --- | --- | --- | --- | --- | --- | --- | --- |
|  | ß | 95% CI | *p* | ß | 95% CI | *p* | ß | 95% CI | *p* | ß | 95% CI | *p* |
| Model 1: CRF change by group  (ref = control) | -0.002 | -0.02, 0.01 | 0.79 | 0.001 | -0.02, 0.02 | 0.91 | -0.001 | -0.01, 0.01 | 0.83 | 0.001 | -0.01, 0.01 | 0.75 |
| Model 2: CRF change^a^ | 0.18 | -0.21, 0.57 | 0.36 | 0.22 | -0.16, 0.59 | 0.26 | -0.03 | -0.2, 0.14 | 0.71 | 0.02 | -0.19, 0.22 | 0.88 |

Abbreviations: CI = confidence interval, CRF = cardiorespiratory fitness

^a^ Model 2 did not contain an interaction term between CRF change and intervention group

***Sensitivity analysis: complete-cases***

Tables 3 and 4 show the model terms and estimates for, respectively cognitive and mental health outcomes on complete-cases only. CRF assessments at post-test were completed during summer term only (for the available-case dataset), hence this confound was excluded from the models. The interaction of change in CRF by intervention group, nor the main effect of change in CRF were significantly related to changes in outcome measures.

**Table 3. Models examining whether a change in CRF is related to a change in cognitive outcomes**

|  | **Relational memory (n = 1791)** | | | **Processing speed (n = 3212)** | | |
| --- | --- | --- | --- | --- | --- | --- |
|  | ß | 95% CI | *p* | ß | 95% CI | *p* |
| Model 1: CRF change by group (ref = control) | 0.015 | -0.081, 0.111 | 0.75 | -0.418 | -1.101, 0.266 | 0.22 |
| Model 2: CRF change ^a^ | 0.01 | -0.041, 0.062 | 0.68 | -0.043 | -0.4, 0.314 | 0.81 |

Abbreviations: CI = confidence interval, CRF = cardiorespiratory fitness

^a^ Model 2 did not contain an interaction term between CRF change and intervention group

**Table 4. Models examining whether a change in CRF is related to a change in mental health outcomes**

|  | **Internalising symptoms**  **(n = 3490)** | | | **Externalising symptoms**  **(n = 3486)** | | | **Global self-esteem**  **(n = 3394)** | | | **Physical self-esteem**  **(n = 3395)** | | |
| --- | --- | --- | --- | --- | --- | --- | --- | --- | --- | --- | --- | --- |
|  | ß | 95% CI | *p* | ß | 95% CI | *p* | ß | 95% CI | *p* | ß | 95% CI | *p* |
| Model 1: CRF change by  group (ref = control) | 0.011 | -0.014, 0.035 | 0.37 | 0.004 | -0.01, 0.018 | 0.55 | 0 | -0.005, 0.005 | 0.90 | -0.002 | -0.009, 0.005 | 0.5 |
| Model 2: CRF change^a^ | -0.002 | -0.013, 0.008 | 0.65 | -0.003 | -0.009, 0.004 | 0.38 | 0.002 | 0, 0.005 | 0.06 | 0.002 | -0.001, 0.005 | 0.2 |

Abbreviations: CI = confidence interval, CRF = cardiorespiratory fitness

^a^ Model 2 did not contain an interaction term between CRF change and intervention group
