# Supplementary material for: The effect of a one-year vigorous physical activity intervention on fitness, cognitive performance and mental health in young adolescents: the Fit to Study cluster randomised controlled trial
Source: Int J Behav Nutr Phys Act. 2021 Mar 31;18:47. doi: 10.1186/s12966-021-01113-y (PMC8011147; doi:10.1186/s12966-021-01113-y)
Supplement: Supplementary file 11 — Additional file 11:. Intervention fidelity and complier-average causal effect (CACE) analysis [file 12966_2021_1113_MOESM11_ESM.docx]

**ADDITIONAL FILE 11. Intervention fidelity and CACE analysis**

Table 1 presents school level fidelity measures collected at different times throughout the trial. In particular, teacher-reported compliance was collected post-intervention by NatCen Social Research using a survey asking intervention schools about the percentage of PE lessons in which the intervention was delivered as intended. Only a subset of 22 (of 29 still enrolled at post-intervention) intervention schools completed this survey. Pupil reported compliance with the intervention was collected using a brief three-question survey administered post-intervention within the online questionnaire asking whether (i) PE lessons started with a warm-up [1 = never to 5 = always], (ii) PE lessons included bursts of VPA that raised their heart rate and made them feel out of breath (i.e. infusions) [1 = never to 5 = always], and (iii) they took part in warm-ups or VPA bursts if asked by PE teachers (i.e. whether they “participated”) [1 = never to 5 = always]. Given that the intervention was delivered on a school level and the questionnaire was completed by subsets of pupils within schools, we computed median values of questionnaire items within schools and present averages and median values across schools. In addition to self-report measures of fidelity, we collected an objective measure of VPA per hour of PE using the Axivity AX3 accelerometer, in a convenience sample of participants per school (at least 50% of the Year group). Data was collected during a single PE lesson between March – May 2018. The total minutes spent in VPA per hour of PE, with or without adjustment for change time (resp. full lesson vs active lesson) were computed per class and averaged within a school. Finally, Table 1 contains information on whether PE teachers of intervention schools attended teacher training sessions face-to-face, online or neither (i.e. did not attend any session). Schools of which PE teachers did not attend any teacher training sessions (n = 5) were lost to follow-up.

Of 46 intervention schools, 17 (37%) implemented the intervention in over 50% of PE lessons during the year. Median and average pupil reported warm ups, infusions and participation on a school level are comparable across intervention and control schools. Average minutes spent in VPA per active hour of PE was slightly higher in intervention schools (M [SD] = 4.91 [1.77]) compared to control schools (M [SD] = 4.38 [1.93]).

**Table 1. School level fidelity measures**

|  | **Intervention (n = 46)** | **Control (n = 47)** | ***P*^b^** |
| --- | --- | --- | --- |
| **Teacher reported compliance (NatCen), no (%)** |  |  |  |
| 0-10% of PE lessons | 1 (2.2%) |  |  |
| 10-25% of PE lessons | 1 (2.2%) |  |  |
| 25-50% of PE lessons | 3 (6.5%) |  |  |
| 50-75% of PE lessons | 8 (17.4%) |  |  |
| 75-90% of PE lessons | 7 (15.2%) |  |  |
| 90-100% of PE lessons | 2 (4.3%) |  |  |
| Missing, no. (%) | 24 (52.2%) | 47 (100%) |  |
| **Pupil reported warm-up (school medians)^1^** |  |  |  |
| Mean (SD) | 4.39 (0.533) | 4.17 (0.747) | 0.21 |
| Median (range) | 4 (3.5-4) | 4 (3-5) |  |
| Missing | 24 (52.2%) | 12 (25.5%) |  |
| **Pupil reported infusions (school medians)^a^** |  |  |  |
| Mean (SD) | 3.91 (0.294) | 3.83 (0.453) | 0.42 |
| Median (range) | 4 (3-4) | 4 (3-5) |  |
| Missing, no. (%) | 24 (52.2%) | 12 (25.5%) |  |
| **Pupil reported participation (school medians)^a^** |  |  |  |
| Mean (SD) | 4.52 (0.499) | 4.49 (0.562) | 0.8 |
| Median (range) | 4.75 (4-5) | 5 (3-5) |  |
| Missing, no. (%) | 24 (52.2%) | 12 (25.5%) |  |
| **VPA (PE lesson, school average)** |  |  |  |
| Full lesson, mean (SD), min | 3.65 (1.09) | 3.09 (1.43) | 0.23 |
| Active part, mean (SD), min | 4.91 (1.77) | 4.38 (1.93) | 0.07 |
| Missing | 16 (34.8%) | 6 (12.8%) |  |
| **Teacher training** |  |  |  |
| Face-to-face, no. (%) | 15 (32.6%) |  |  |
| Online, no. (%) | 26 (56.5%) |  |  |
| None, no. (%) | 5 (10.9%) | 47 (100%) |  |

Abbreviations: No = number, PE = physical education, SD = standard deviation, VPA = vigorous physical activity

^a^ Median across pupils within a school. Measures were based on 5-point Likert-scales, ranging from never = 1 to always = 5

^b^ Assessed using unpaired t-tests, uncorrected for multiple testing.

**CACE analysis**

The complier average causal effect was determined using a two-stage least squares (TSLS) method. Compliers were defined as schools that delivered the intervention > 50% of PE lessons. The CACE was first estimated on the multiply imputed sample (Table 2) and then on the complete-case sample (Table 3). CACE estimates were comparable in direction, though slightly larger in size, to ITT estimates provided in the main manuscript file. None of the analyses demonstrated evidence for a significant complier-average causal effect.

**Table 2. CACE estimates and confidence intervals (multiply imputed data)**

|  | **CACE (unstandardized)** | **95% CI** |
| --- | --- | --- |
| 20MSR |  |  |
| Fitness, laps | 0.03 | -8.05, 8.11 |
| Reaction time task |  |  |
| RT, ms | 4.44 | -22.54, 31.43 |
| Relational memory task |  |  |
| Accuracy, % | -3.46 | -7.95, 1.02 |
| Two-back task |  |  |
| Accuracy, % | -2.1 | -8.18, 3.99 |
| RT, ms | -9.11 | -56.74, 38.52 |
| Flanker task |  |  |
| Accuracy congruent, % | -2.21 | -6.31, 1.9 |
| Accuracy incongruent, % | -3.3 | -8.46, 1.86 |
| RT congruent, ms | -9.41 | -31.34, 12.52 |
| RT incongruent, ms | -16.87 | -42.79, 9.04 |
| Colour-shape switching task |  |  |
| Accuracy non-switch, % | -1.75 | -6.59, 3.09 |
| Accuracy switch, % | -0.76 | -5.59, 4.07 |
| RT non-switch, ms | -16.86 | -96.72, 63 |
| RT switch, ms | -50.33 | -198.68, 98.02 |
| Psychosocial problems |  |  |
| Internalising score | 0.44 | -0.63, 1.51 |
| Externalising score | 0.67 | -0.42, 1.75 |
| Self-esteem |  |  |
| Global | -0.16 | -0.73, 0.41 |
| Physical | -0.09 | -0.71, 0.52 |

Abbreviations: 20MSR = 20 meter shuttle run, ms = millisecond, RT = reaction time

**Table 3. CACE estimates and confidence intervals (complete-case data)**

|  | **CACE (unstandardized)** | **95% CI** |
| --- | --- | --- |
| 20MSR |  |  |
| Fitness, laps | 1.84 | -2.85, 6.54 |
| Reaction time task |  |  |
| RT, ms | 11.7 | -11.05, 34.45 |
| Relational memory task |  |  |
| Accuracy, % | -2.15 | -5.5, 1.21 |
| Two-back task |  |  |
| Accuracy, % | -1.83 | -7.06, 3.4 |
| RT, ms | 2.94 | -33.31, 39.19 |
| Flanker task |  |  |
| Accuracy congruent, % | -0.79 | -3.32, 1.75 |
| Accuracy incongruent, % | -1.56 | -4.84, 1.72 |
| RT congruent, ms | -2.14 | -12.93, 8.66 |
| RT incongruent, ms | -7.84 | -20.05, 4.36 |
| Colour-shape switching task |  |  |
| Accuracy non-switch, % | -2.27 | -6.01, 1.48 |
| Accuracy switch, % | -0.95 | -4.35, 2.46 |
| RT non-switch, ms | 15.67 | -38.57, 69.91 |
| RT switch, ms | -23.53 | -119.94, 72.87 |
| Psychosocial problems |  |  |
| Internalising score | 0.02 | -0.36, 0.39 |
| Externalising score | 0.04 | -0.35, 0.44 |
| Self-esteem |  |  |
| Global | 0.01 | -0.09, 0.11 |
| Physical | -0.02 | -0.11, 0.07 |

Abbreviations: 20MSR = 20 meter shuttle run, ms = millisecond, RT = reaction time
