# Supplementary material for: The effect of a one-year vigorous physical activity intervention on fitness, cognitive performance and mental health in young adolescents: the Fit to Study cluster randomised controlled trial
Source: Int J Behav Nutr Phys Act. 2021 Mar 31;18:47. doi: 10.1186/s12966-021-01113-y (PMC8011147; doi:10.1186/s12966-021-01113-y)
Supplement: Supplementary file 12 — Additional file 12:. Additional analyses of PE enjoyment and attitudes towards PA [file 12966_2021_1113_MOESM12_ESM.docx]

**Additional file 12. Additional analysis**

We examined whether there was a difference in PE enjoyment or attitudes towards PA between the intervention and control group at baseline (Table 1) and at posttest (Table 2) on multiply imputed data and using complete-case data (Tables 3 and 4) as a sensitivity analysis. No significant differences between the groups were observed.

**Table 1. Models examining baseline differences in PE enjoyment and attitudes towards PA**

|  | **Intervention** | **Control** | **Adjusted mean difference^1^ (95% CI)** | | ***p*** |
| --- | --- | --- | --- | --- | --- |
|  | M (SD) | M (SD) | Unstandardized | Standardized^2^ |  |
| PE enjoyment | 5.81 (1.61) | 5.81 (1.59) | 0.01 (-0.14, 0.16) | 0.01 (-0.09, 0.1) | 0.91 |
| Attitudes towards PA | 4.97 (1.28) | 5.01 (1.28) | -0.03 (-0.15, 0.08) | -0.03 (-0.12, 0.07) | 0.57 |

Abbreviations: CI = confidence interval, M = mean, PA = physical activity, PE = physical education, SD = standard deviation

^1^Models contained the intervention group and were adjusted for clustering and school-gender type

^2^Outcomes were scaled (mean = 0, SD = 1) prior to running the model

**Table 2. Models examining the effect of the intervention on PE enjoyment and attitudes towards PA**

|  | **Intervention** | | **Control** | | **Adjusted mean difference^1^ (95% CI)** | | ***p*** |
| --- | --- | --- | --- | --- | --- | --- | --- |
|  | Baseline  M(SD) | Post  M(SD) | Baseline  M(SD) | Post  M(SD) | Unstandardized | Standardized^2^ |  |
| PE enjoyment | 5.81 (1.61) | 5.49 (1.73) | 5.81 (1.59) | 5.56 (1.69) | -0.06 (-0.28, 0.16) | -0.04 (-0.16, 0.09) | 0.58 |
| Attitudes towards PA | 4.97 (1.28) | 4.52 (1.34) | 5.01 (1.28) | 4.7 (1.34) | -0.15 (-0.34, 0.03) | -0.11 (-0.25, 0.02) | 0.1 |

Abbreviations: CI = confidence interval, M = mean, PA = physical activity, PE = physical education, SD = standard deviation

^1^Models contained the intervention group and were adjusted for clustering and school-gender type

^2^Outcomes were scaled (mean = 0, SD = 1) prior to running the model

***Sensitivity analysis: complete-cases***

We examined whether there was a difference in PE enjoyment or attitudes towards PA between the intervention and control group at baseline (Table 3) and at posttest (Table 4). No significant differences between the groups were observed.

**Table 3. Models examining baseline differences in PE enjoyment and attitudes towards PA.**

|  | **Intervention** | | **Control** | | **Adjusted mean difference (95% CI)^1^** | | ***p*** |
| --- | --- | --- | --- | --- | --- | --- | --- |
|  | N | M (SD) | N | M (SD) | Unstandardized | Standardized^2^ |  |
| PE enjoyment | 4734 | 5.78 (1.59) | 5040 | 5.77 (1.57) | 0.01 ( -0.12, 0.14) | 0.01 ( -0.08, 0.09) | 0.81 |
| Attitudes towards PA | 4734 | 4.97 (1.26) | 5040 | 4.98 (1.27) | -0.01 (-0.11, 0.09) | -0.01 (-0.09, 0.07) | 0.85 |

Abbreviations: CI = confidence interval, M = mean, PA = physical activity, PE = physical education, SD = standard deviation

^1^Models contained the intervention group and were adjusted for clustering and school-gender type

^2^Outcomes were scaled (mean = 0, SD = 1) prior to running the model

**Table 4. Models examining the effect of the intervention on PE enjoyment and attitudes towards PA**

|  | **Intervention** | | | **Control** | | | **Adjusted mean difference^1^ (95% CI)** | | ***p*** |
| --- | --- | --- | --- | --- | --- | --- | --- | --- | --- |
|  | N | Baseline  M(SD) | Post  M(SD) | N | Baseline  M(SD) | Post  M(SD) | Unstandardized | Standardized^2^ |  |
| PE enjoyment | 1899 | 5.76 (1.59) | 5.49 (1.64) | 2797 | 5.76 (1.54) | 5.54 (1.65) | -0.02 (-0.14, 0.09) | -0.01 (-0.08, 0.06) | 0.69 |
| Attitudes towards PA | 1899 | 4.97 (1.25) | 4.54 (1.26) | 2797 | 5.02 (1.23) | 4.65 (1.31) | -0.07 (-0.16, 0.01) | -0.06 (-0.12, 0.01) | 0.08 |

Abbreviations: CI = confidence interval, M = mean, PA = physical activity, PE = physical education, SD = standard deviation

^1^Models contained the intervention group and were adjusted for clustering and school-gender type

^2^Outcomes were scaled (mean = 0, SD = 1) prior to running the model
